# Supplementary material for: Regionalized regulation of actomyosin organization influences cardiomyocyte cell shape changes during chamber curvature formation
Source: Nat Commun. 2026 Mar 10;17:3768. doi: 10.1038/s41467-026-70384-5 (PMC13106787; doi:10.1038/s41467-026-70384-5)
Supplement: Supplementary file 1 — Supplementary Information [file 41467_2026_70384_MOESM1_ESM.pdf]

Supplementary Information

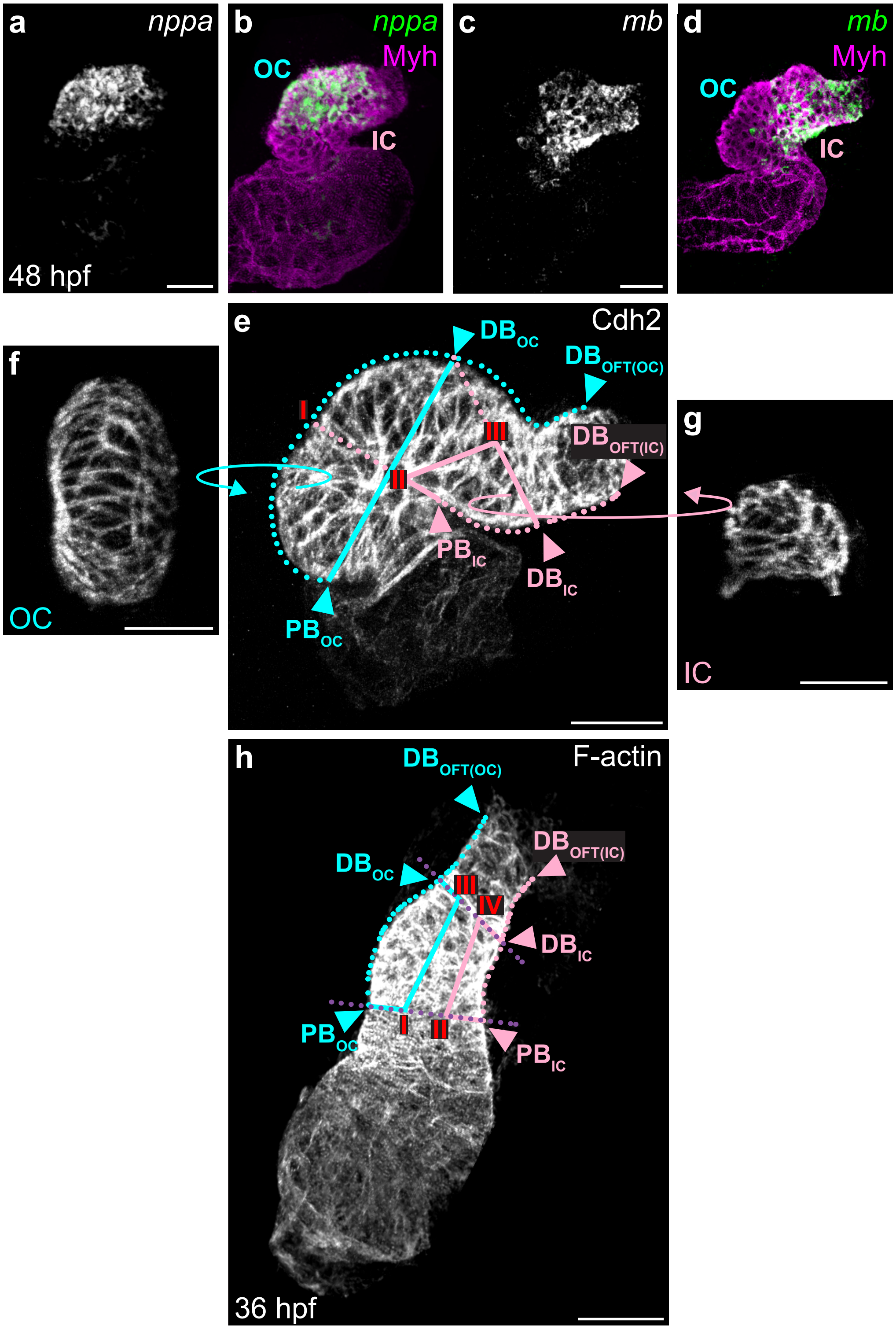

Supplementary Figure 1. Defining the ventricular OC and IC.

(**a,c**) Fluorescent *in situ* hybridization (FISH) for *nppa* (a) and *mb* (*myoglobin*, c) in 48 hpf wild-type hearts. (**b,d**) FISH signal is merged with immunostaining for Myosin heavy chain (Myh), outlining the entire myocardium. *nppa* is enriched primarily in the OC of the ventricle, and *mb* is enriched in the ventricular IC and the outflow tract (OFT). (**e**) 3D reconstruction of a 48 hpf wild-type heart immunostained for Cdh2, which labels the lateral membranes of cardiomyocytes. Lines represent measurements used to segment the OC and IC (refer to Methods for more detail, including information on the red "I", "II", and "III"). Once boundaries have been drawn in (e), the resulting OC (**f**) and IC (**g**) are swiveled to be viewed *en face*. (**h**) 3D reconstruction of a 36 hpf wild-type heart stained with Phalloidin, outlining F-actin distribution throughout the entire heart. Lines represent measurements used to delineate the OC and IC (refer to Methods for more detail, including information on the red "I", "II", "III", and "IV"). PB_{OC}: OC proximal boundary. DB_{OC}: OC distal boundary. $DB_{OFT(OC)}$: distal boundary of the OC side of the OFT. PB_{IC}: IC proximal boundary. DB_{IC}: IC distal boundary. $DB_{OFT(IC)}$: distal boundary of the IC side of the OFT. *nppa* (three experimental replicates, N=12 embryos); *mb* (two experimental replicates, N=6 embryos). Scale bars = 40 µm (a,c,e-g); 50 µm (h).

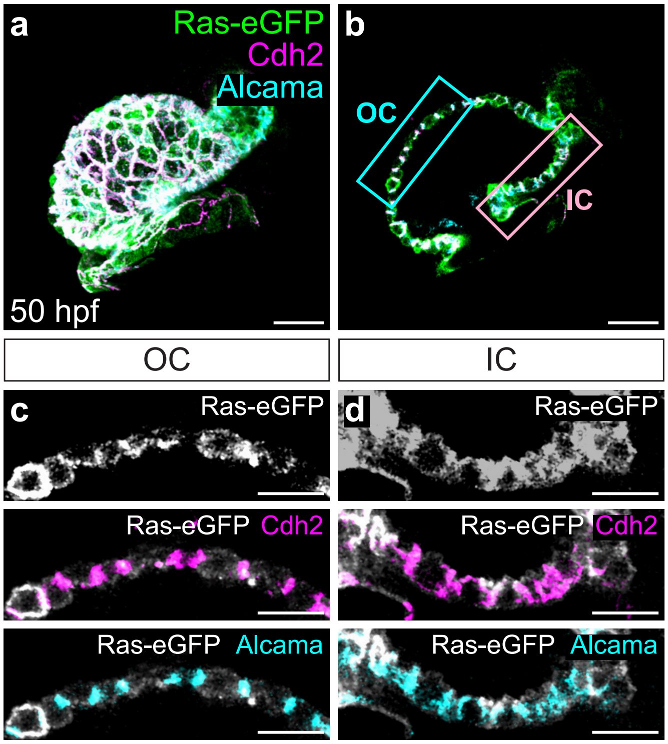

Supplementary Figure 2. Comparing lateral membrane markers.

(**a,b**) Heart from a 50 hpf embryo carrying *Tg(myl7:eGFP-Hsa.HRAS)* [61], immunostained for membrane-bound eGFP and for cell adhesion molecules Cdh2 and Alcama. (b) shows a section through the 3D reconstruction shown in (a). (**c,d**) Magnified views of blue (OC) and pink (IC) boxed regions in (b). Colocalization of Cdh2 and Alcama signals with the Ras-eGFP signal at the lateral membranes highlights the ability of these markers to label the entire lateral membrane. Data represent one experimental replicate, N=8 embryos. Scale bars = 30 µm (a,b), 15 µm (c,d).

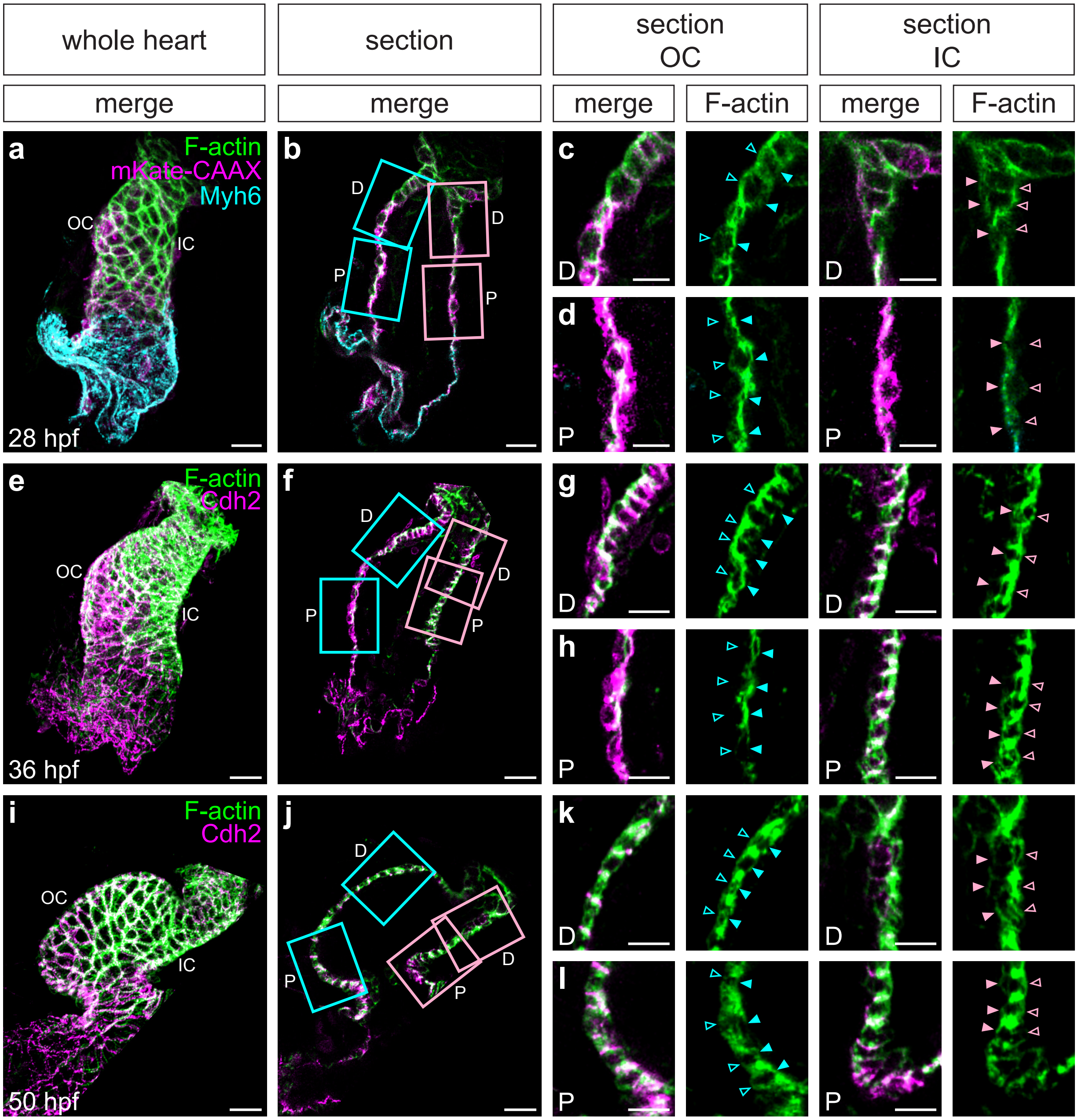

whole heart
section
section OC
section IC
merge
F-actin
a
F-actin
mKate-CAAX
Myh6
OC
IC
28 hpf
b
D
P
c
d
e
F-actin
Cdh2
OC
IC
36 hpf
f
g
h
i
F-actin
Cdh2
OC
IC
50 hpf
j
k
l

Supplementary Figure 3. Regionalized subcellular F-actin localization changes as curvatures form.

(**a**) Whole heart from a 28 hpf embryo carrying *Tg(myl7:mKate-CAAX)* [62], immunostained for membrane-bound mKate and for Myh6 to determine the atrial boundary. (**e,i**) Whole hearts from 36 hpf (e) and 50 hpf (i) embryos immunostained for Cdh2 to label lateral membranes of cardiomyocytes. All hearts stained with Phalloidin to label F-actin. (**b,f,j**) Sections through hearts in (a,e,i). (**c,d,g,h,k,l**) Magnified views of blue (OC) and pink (IC) boxed sections in (b,f,j); both distal (D) and proximal (P) regions are shown. Empty arrowheads: apical membranes. Filled arrowheads: basal membranes. Data represent two experimental replicates. 28 hpf (N=11 embryos); 36 hpf (N=15 embryos); 50 hpf (N=10 embryos). Scale bars = 20 µm (a,b,e,f,i,j), 10 µm (c,d,g,h,k,l).

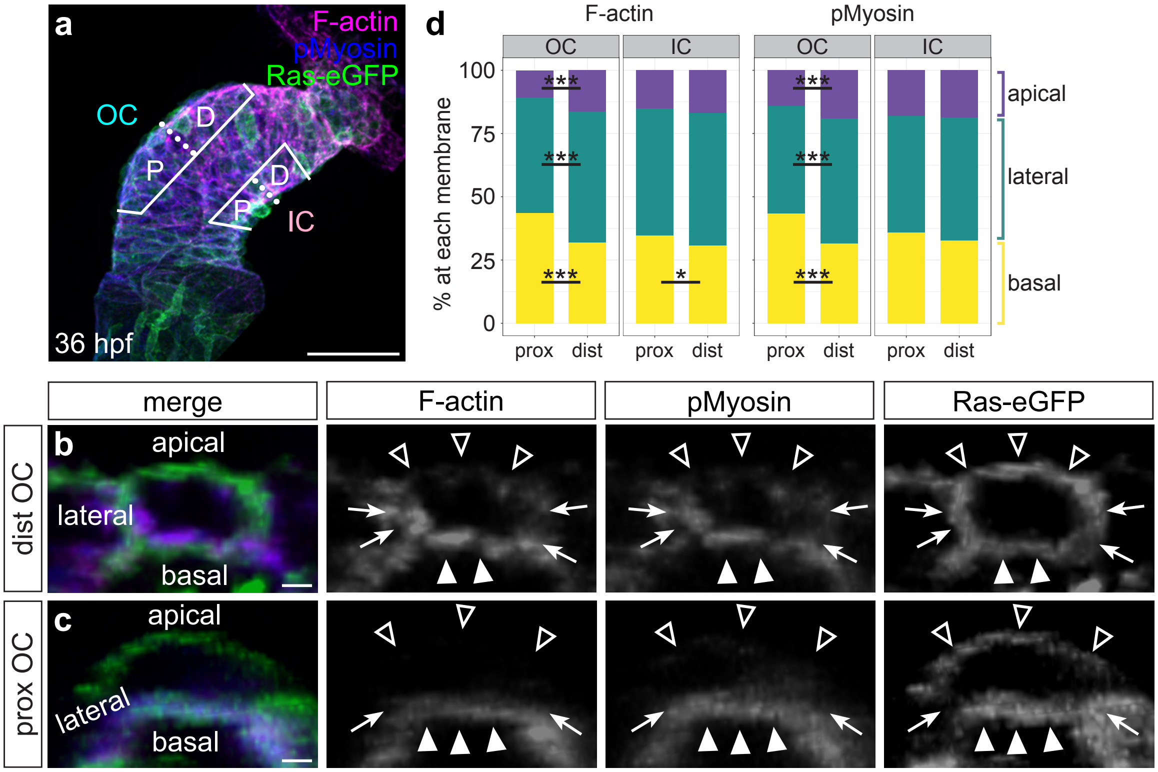

Supplementary Figure 4. Differences in actomyosin localization along the proximodistal axis of the OC.

(**a**) Whole heart from a 36 hpf embryo carrying *Tg(myl7:eGFP-Hsa.HRAS)*, immunostained for membrane-bound GFP and pMyosin and stained with Phalloidin to label F-actin (as in Fig. 2a). Solid lines outline the OC and IC; dotted lines show division between the proximal (P) and distal (D) halves of each curvature. (**b,c**) Cross-sections through representative individual cardiomyocytes from the distal OC (b) or proximal OC (c). Empty arrowheads: apical membranes. Filled arrowheads: basal membranes. Arrows: lateral membranes. (**d**) Stacked bar charts showing the mean percentage of F-actin or pMyosin at each membrane. Refer to Supplementary Tables 3 and 4 for summary statistics. For ratiometric depiction of these data, see Supplementary Fig. 5. Two-sided Wilcoxon test. For F-actin in (d), proximal OC vs distal OC (basal): $p = 5.05*10^{-13}$; proximal OC vs distal OC (lateral): $p = 4.91*10^{-7}$; proximal OC vs distal OC (apical): $p = 2.78*10^{-10}$; proximal IC vs distal IC (basal): $p = 0.0204$. For pMyosin in (d), proximal OC vs distal OC (basal): $p = 2.08*10^{-10}$; proximal OC vs distal OC (lateral): $p = 1.40*10^{-7}$; proximal OC vs distal OC (apical): $p = 3.92*10^{-7}$. Data represent two experimental

replicates. N=10 embryos; proximal OC (n=97 cells); distal OC (n=102 cells); proximal IC (n=63 cells); distal IC (n=60 cells). Scale bars = 50 μm (a), 2 μm (b,c).

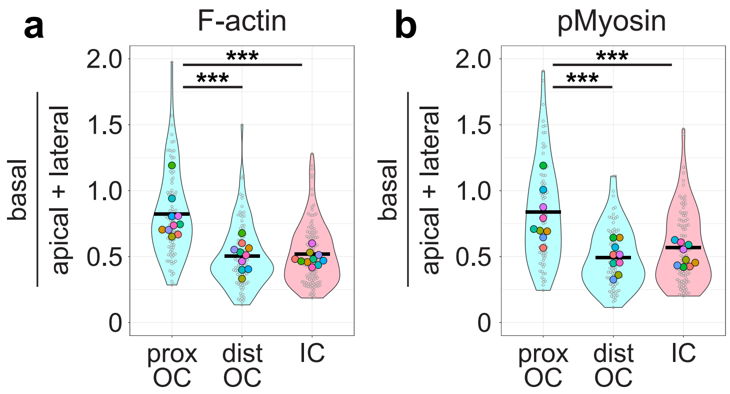

Supplementary Figure 5. Ratiometric comparison of basal to apical and lateral actomyosin highlights basal enrichment of actomyosin along the proximodistal axis of the OC.

(**a**) Violin plot depicts data from Supplementary Fig. 4, recalculated as (mean basal F-actin / (mean apical + mean lateral F-actin)) for individual cells. (**b**) Violin plot of recalculated values as in (a), but for pMyosin. Each small grey dot represents an individual cell, each black bar represents the mean of values from individual cells, and each large colored dot represents the mean of all values from an individual embryo. Two-sided Wilcoxon test. (a) proximal OC vs distal OC: $p = 5.05*10^{-13}$; proximal OC vs IC: $p = 3.21*10^{-13}$. (b) proximal OC vs distal OC: $p = 2.08*10^{-10}$; proximal OC vs IC: $p = 1.73*10^{-7}$. Data represent two experimental replicates. N=10 embryos; proximal OC (n=97 cells); distal OC (n=102 cells); IC (n=123 cells).

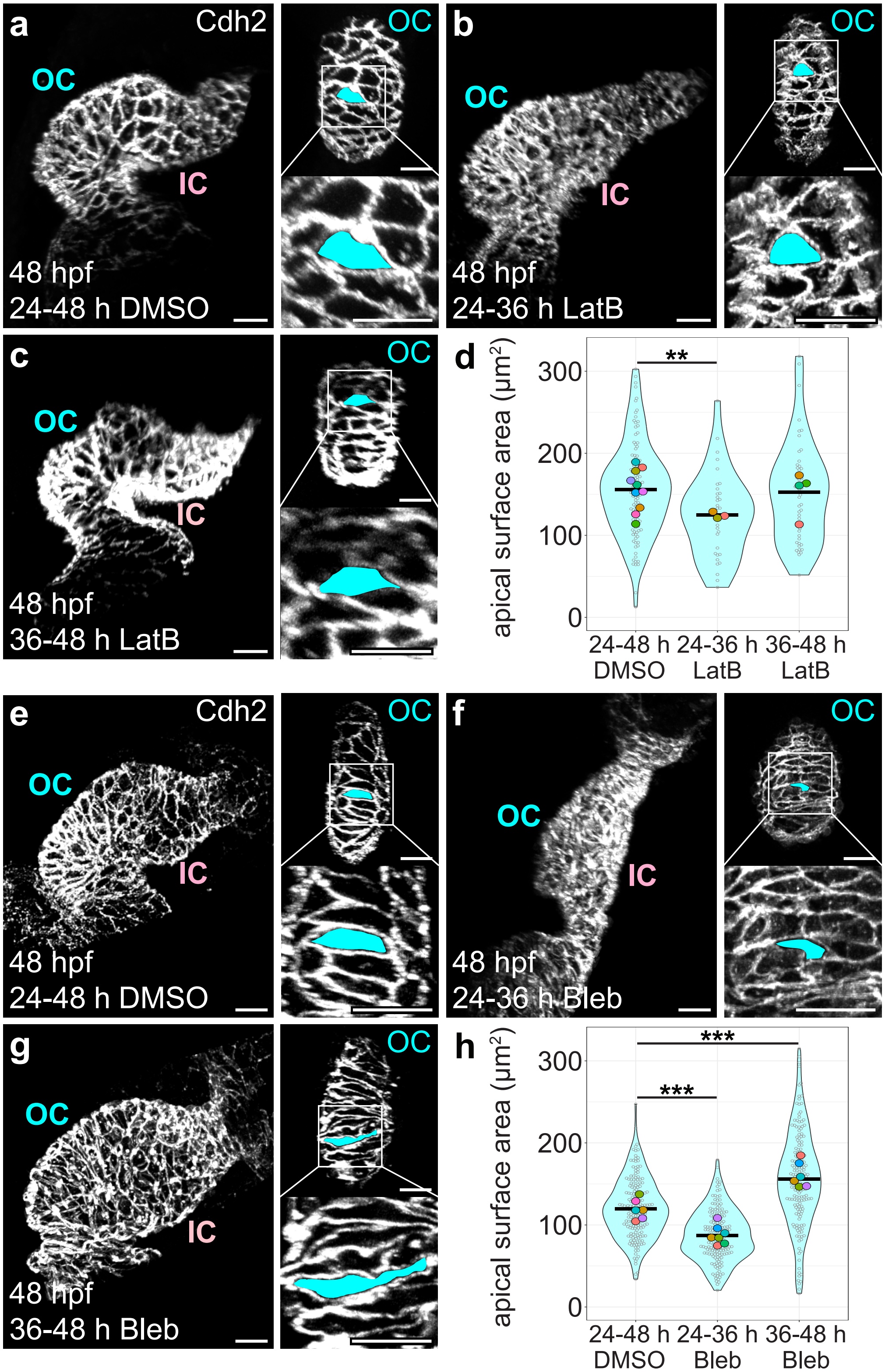

a
Cdh2
OC
IC
48 hpf
24-48 h DMSO
b
OC
IC
48 hpf
24-36 h LatB
c
OC
IC
48 hpf
36-48 h LatB
d
apical surface area (μm²)
**
300
200
100
0
24-48 h DMSO
24-36 h LatB
36-48 h LatB
e
Cdh2
OC
IC
48 hpf
24-48 h DMSO
f
OC
IC
48 hpf
24-36 h Bleb
g
OC
IC
48 hpf
36-48 h Bleb
h
apical surface area (μm²)
***
***
300
200
100
0
24-48 h DMSO
24-36 h Bleb
36-48 h Bleb

Supplementary Figure 6. Pharmacological inhibition of actin polymerization or NMII activity during early curvature formation disrupts the acquisition of characteristic OC cardiomyocyte morphology.

(**a-c,e-g**) 3D reconstructions of 48 hpf wild-type hearts and their OCs, from embryos treated with DMSO from 24-48 hpf (a,e), 100 ng/mL Latrunculin B (Lat B) from 24-36 hpf (b), 100 ng/mL Lat B from 36-48 hpf (c), 5 uM Blebbistatin (Bleb) from 24-36 hpf (f), or 5 uM Bleb from 36-48 hpf (g). Immunostaining for Cdh2 labels lateral membranes of cardiomyocytes. Insets show higher magnification of OC cells. Apical surface area of an individual cardiomyocyte is illustrated by blue fill. (**d,h**) Violin plots compare apical surface area of OC cells following different treatments. Each small grey dot represents an individual cell, each black bar represents the mean of values from individual cells, and each large colored dot represents the mean of all values from an individual embryo. Two-sided Wilcoxon test. (d) $p = 0.00317$. (h) DMSO vs 24-36 hpf Bleb: $p = 6.32*10^{-17}$; DMSO vs 36-48 hpf Bleb: $p = 4.75*10^{-12}$. Data represent one experimental replicate. For Lat B: DMSO 24-48 hpf (N=10 embryos, n=120 cells); Lat B 24-36 hpf (N=3 embryos, n=35 cells); Lat B 36-48 hpf (N=4 embryos, n=48 cells). For Bleb: DMSO 24-48 hpf (N=6 embryos, n=183 cells); Lat B 24-36 hpf (N=7 embryos, n=169 cells); Lat B 36-48 hpf (N=6 embryos, n=170 cells). Scale bars = 20 µm.

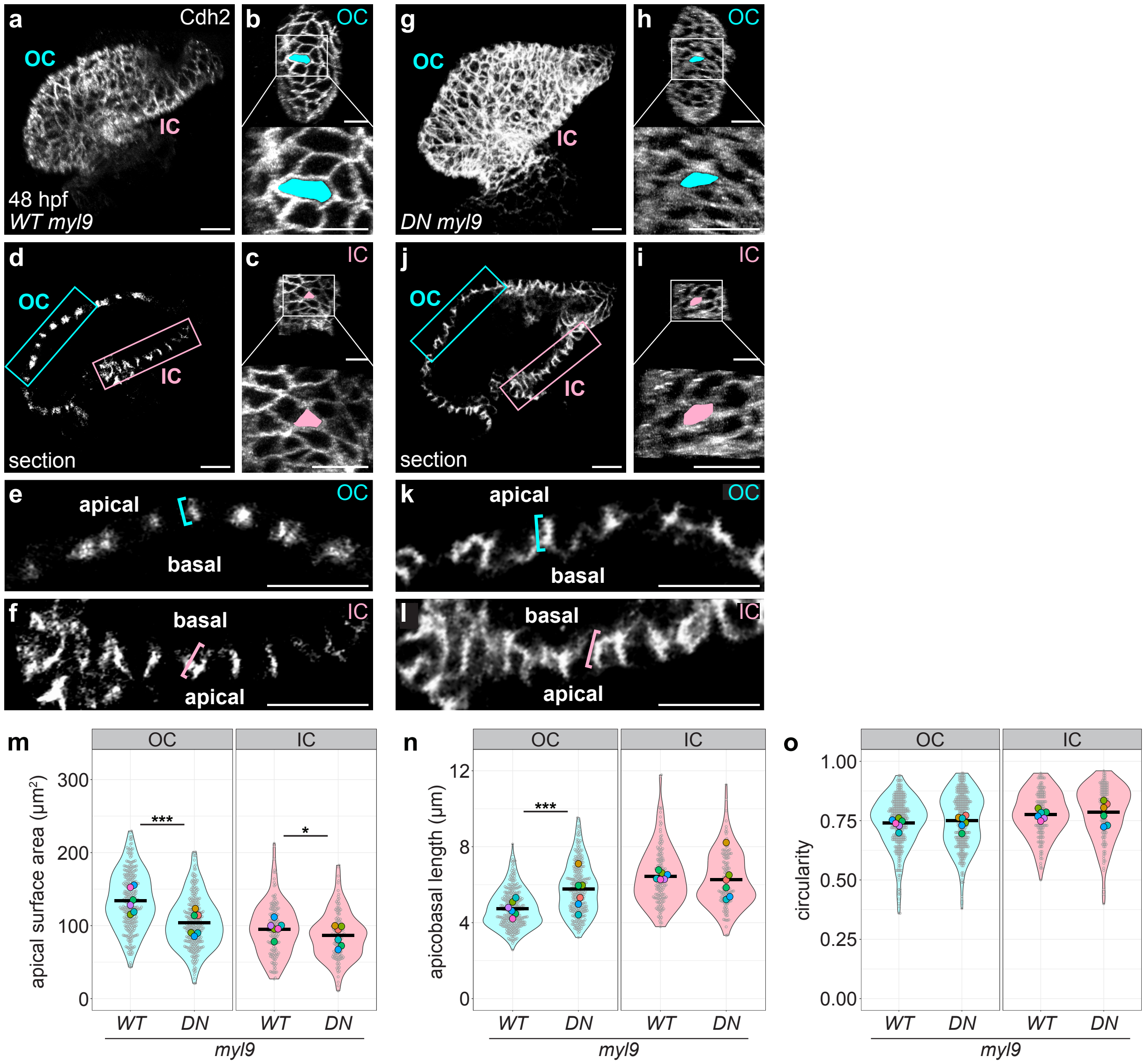

a
Cdh2
OC
IC
48 hpf
WT myl9
b
OC
g
OC
IC
DN myl9
h
OC
d
OC
IC
section
c
IC
j
OC
IC
section
i
IC
e
apical
basal
OC
k
apical
basal
OC
f
basal
apical
IC
l
basal
apical
IC
m
apical surface area (µm²)
OC
IC
***
*
WT
DN
myl9
n
apicobasal length (µm)
OC
IC
***
WT
DN
myl9
o
circularity
OC
IC
WT
DN
myl9

Supplementary Figure 7. Tissue-specific inhibition of NMII activity dampens the divergence of OC and IC cardiomyocyte morphologies.

(**a,g**) 3D reconstructions of 48 hpf hearts expressing *Tg(myl7:WT-myl9-mScarlet)* (a) or *Tg(myl7:DN-myl9-eGFP)* (g). Immunostaining for Cdh2 labels lateral membranes of cardiomyocytes. OCs **(b,h)** and ICs **(c,i)** are shown for hearts in (a) and (g). Insets show higher magnification. Apical surface area of an individual cardiomyocyte is illustrated by blue or pink fill. (**d,j**) Sections through hearts in (a) and (g). (**e,f,k,l**) Magnified views of blue (OC) and pink (IC) boxed regions in (d) and (j); blue and pink brackets highlight apicobasal length of individual cardiomyocytes. (**m-o**) Violin plots compare apical surface area, apicobasal length, or circularity of cardiomyocytes between embryos expressing either transgene, split by curvature. Circularity is calculated as $4\pi(A/P^2)$. Each small grey dot represents an individual cell, each black bar represents the mean of values from individual cells, and each large colored dot represents the mean of all values from an individual embryo. Two-sided Wilcoxon test. (m) *Tg(myl7:WT-myl9-mScarlet)* OC vs *Tg(myl7:DN-myl9-eGFP)* OC: $p = 4.34*10^{-20}$; *Tg(myl7:WT-myl9-mScarlet)* IC vs *Tg(myl7:DN-myl9-eGFP)* IC: $p = 0.0262$. (n) $p = 5.74*10^{-22}$. Data represent two experimental replicates. *Tg(myl7:WT-myl9-mScarlet)* OC (N=6 embryos, n=308 cells); *Tg(myl7:WT-myl9-mScarlet)* IC (N=6 embryos, n=159 cells); *Tg(myl7:DN-myl9-eGFP)* OC (N=6 embryos, n=298 cells); *Tg(myl7:DN-myl9-eGFP)* IC (N=6 embryos, n=143 cells). Scale bars = 20 µm.

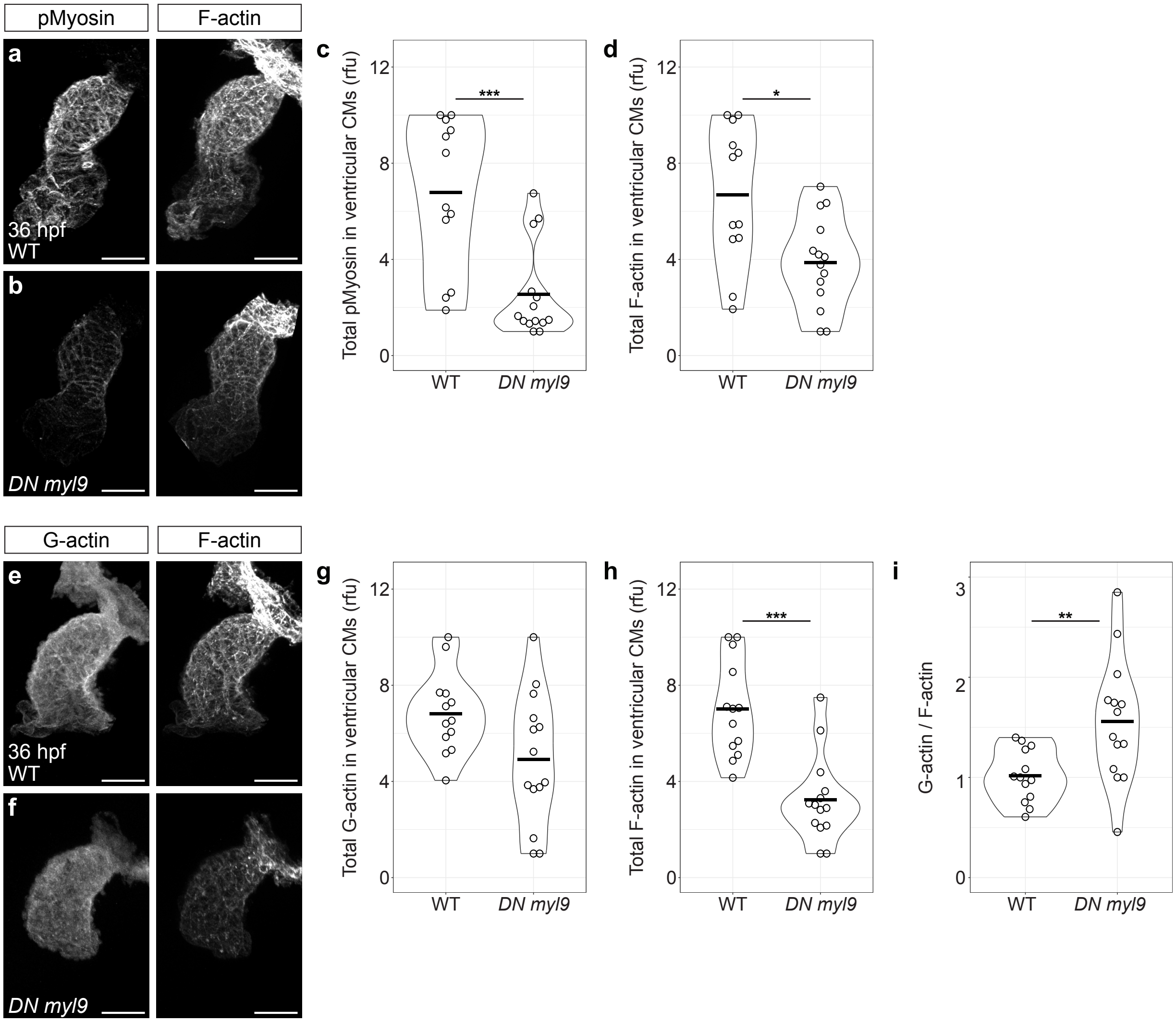
pMyosin
F-actin
a
36 hpf
WT
b
DN myl9
c
Total pMyosin in ventricular CMs (rfu)
***
WT
DN myl9
d
Total F-actin in ventricular CMs (rfu)
*
G-actin
F-actin
e
36 hpf
WT
f
DN myl9
g
Total G-actin in ventricular CMs (rfu)
h
Total F-actin in ventricular CMs (rfu)
***
i
G-actin / F-actin
**

Supplementary Figure 8. Tissue-specific inhibition of NMII activity reduces the total amount of F-actin in the ventricular myocardium.

(**a,b**) 3D reconstructions of a wild-type heart (a) and a heart expressing *Tg(myl7:DN-myl9-eGFP)* (b) at 36 hpf, immunostained for pMyosin and stained with Phalloidin to label F-actin. (**c,d**) Violin plots compare relative fluorescence units (rfu) of pMyosin (c) and F-actin (d) in the ventricular myocardium between wild-type and transgenic embryos. (**e,f**) 3D reconstructions of a wild-type heart (e) and a heart expressing *Tg(myl7:DN-myl9-eGFP)* (f) at 36 hpf, stained with DNase I and Phalloidin to label G-actin and F-actin, respectively. (**g,h**) Violin plots compare rfu of G-actin (g) and F-actin (h) in the ventricular myocardium between wild-type and transgenic embryos. (**i**) Violin plot compares the ratio of G-actin to F-actin between wild-type and transgenic embryos. For (c,d,g-i), each dot represents one ventricle, and each black bar represents the mean. Two-sided Wilcoxon test. (c) $p = 0.000905$. (d) $p = 0.0145$. (h) $p = 0.000248$. (i) $p = 0.00608$. Each graph represents two experimental replicates, and each replicate has undergone normalization to a scale of 1-10 (see Methods for more detail). For (a-d): wild-type (N=12 embryos); *Tg(myl7:DN-myl9-eGFP)* (N=14 embryos). For (e-i): wild-type (N=13 embryos); *Tg(myl7:DN-myl9-eGFP)* (N=14 embryos). Scale bars = 50 µm.

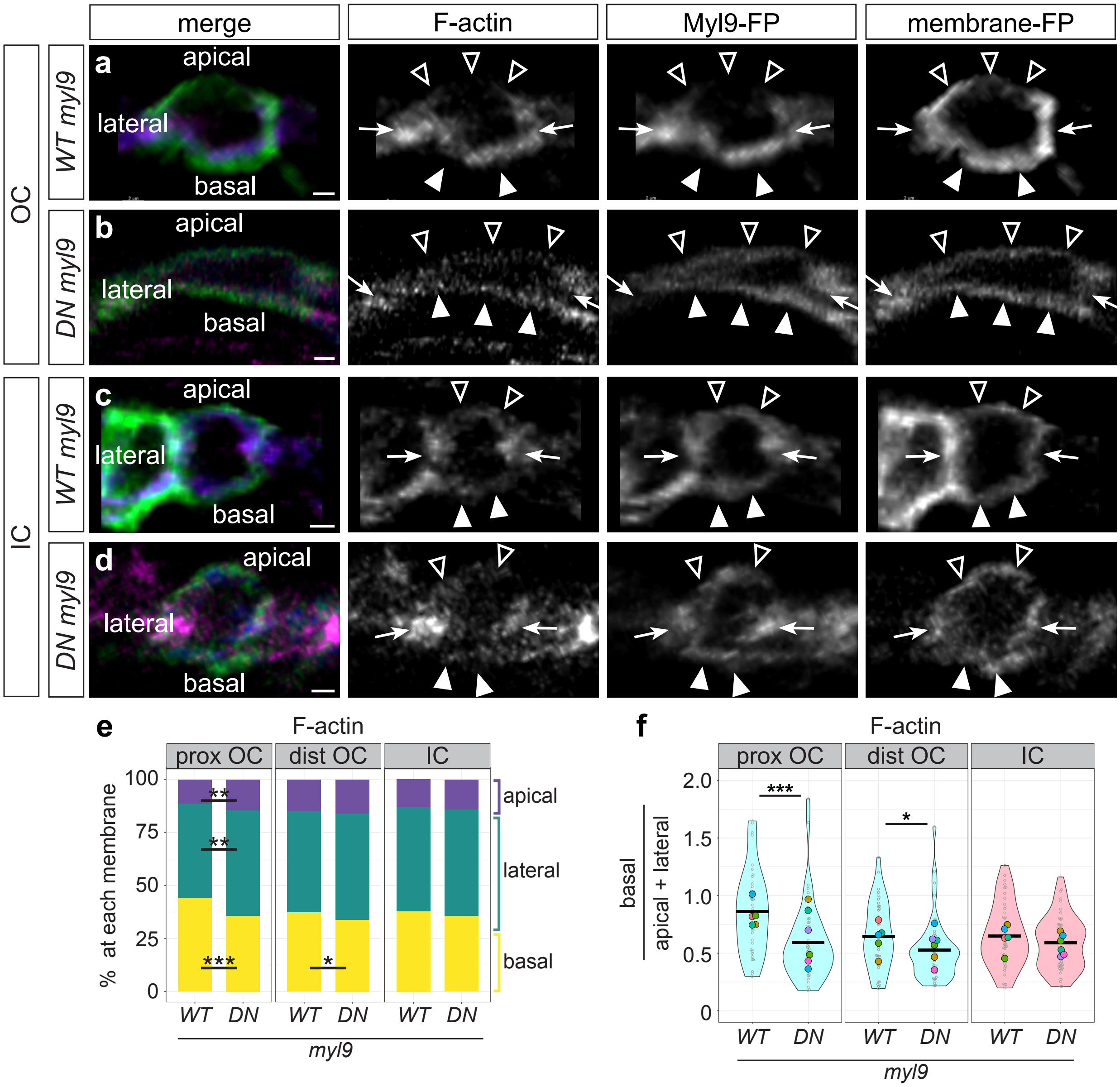
merge
F-actin
Myl9-FP
membrane-FP
OC
IC
a
b
c
d
WT myl9
DN myl9
apical
lateral
basal
e
F-actin
prox OC
dist OC
IC
% at each membrane
100
75
50
25
0
apical
lateral
basal
WT
DN
myl9
f
F-actin
basal
apical + lateral
2.0
1.5
1.0
0.5
0
***
**
*

Supplementary Figure 9. Tissue-specific inhibition of NMII activity alters subcellular F-actin localization in OC cardiomyocytes.

(**a-d**) Cross-sections through representative individual cardiomyocytes from the OC (a,b) or IC (c,d) of 36 hpf embryos expressing *Tg(myl7:WT-myl9-mScarlet)* and *Tg(myl7:eGFP-Hsa.HRAS)* (a,c) or *Tg(myl7:DN-myl9-eGFP)* and *Tg(myl7:mKate-CAAX)* (b,d). All hearts are stained with Phalloidin to label F-actin. In the "merge" column, magenta represents F-actin, blue represents the Myl9-bound fluorescent protein, and green represents the membrane-bound fluorescent protein. Empty arrowheads: apical membranes. Filled arrowheads: basal membranes. Arrows: lateral membranes. (**e**) Stacked bar charts show the mean percentage of F-actin at each membrane. Refer to Supplementary Table 5 for summary statistics. (**f**) Data from (e), recalculated as in Supplementary Fig. 5. Each small grey dot represents an individual cell, each black bar represents the mean of values from individual cells, and each large colored dot represents the mean of all values from an individual embryo. Two-sided Wilcoxon test. (e) *Tg(myl7:WT-myl9-mScarlet)* proximal OC vs *Tg(myl7:DN-myl9-eGFP)* proximal OC (basal): $p = 0.000492$; *Tg(myl7:WT-myl9-mScarlet)* proximal OC vs *Tg(myl7:DN-myl9-eGFP)* proximal OC (lateral): $p = 0.00768$; *Tg(myl7:WT-myl9-mScarlet)* proximal OC vs *Tg(myl7:DN-myl9-eGFP)* proximal OC (apical): $p = 0.00747$; *Tg(myl7:WT-myl9-mScarlet)* distal OC vs *Tg(myl7:DN-myl9-eGFP)* distal OC (basal): $p = 0.0469$. (f) *Tg(myl7:WT-myl9-mScarlet)* proximal OC vs *Tg(myl7:DN-myl9-eGFP)* proximal OC: $p = 0.000492$; *Tg(myl7:WT-myl9-mScarlet)* distal OC vs *Tg(myl7:DN-myl9-eGFP)* distal OC: $p = 0.0469$. Data represent one experimental replicate. For *Tg(myl7:WT-myl9-mScarlet)*: proximal OC (N=5 embryos, n=39 cells); distal OC (N=5 embryos, n=42 cells); IC (N=5 embryos, n=50 cells). For *Tg(myl7:DN-myl9-eGFP)*: proximal OC (N=6 embryos, n=45 cells); distal OC (N=6 embryos, n=46 cells); IC (N=6 embryos, n=53 cells). Scale bars = 2 μm.

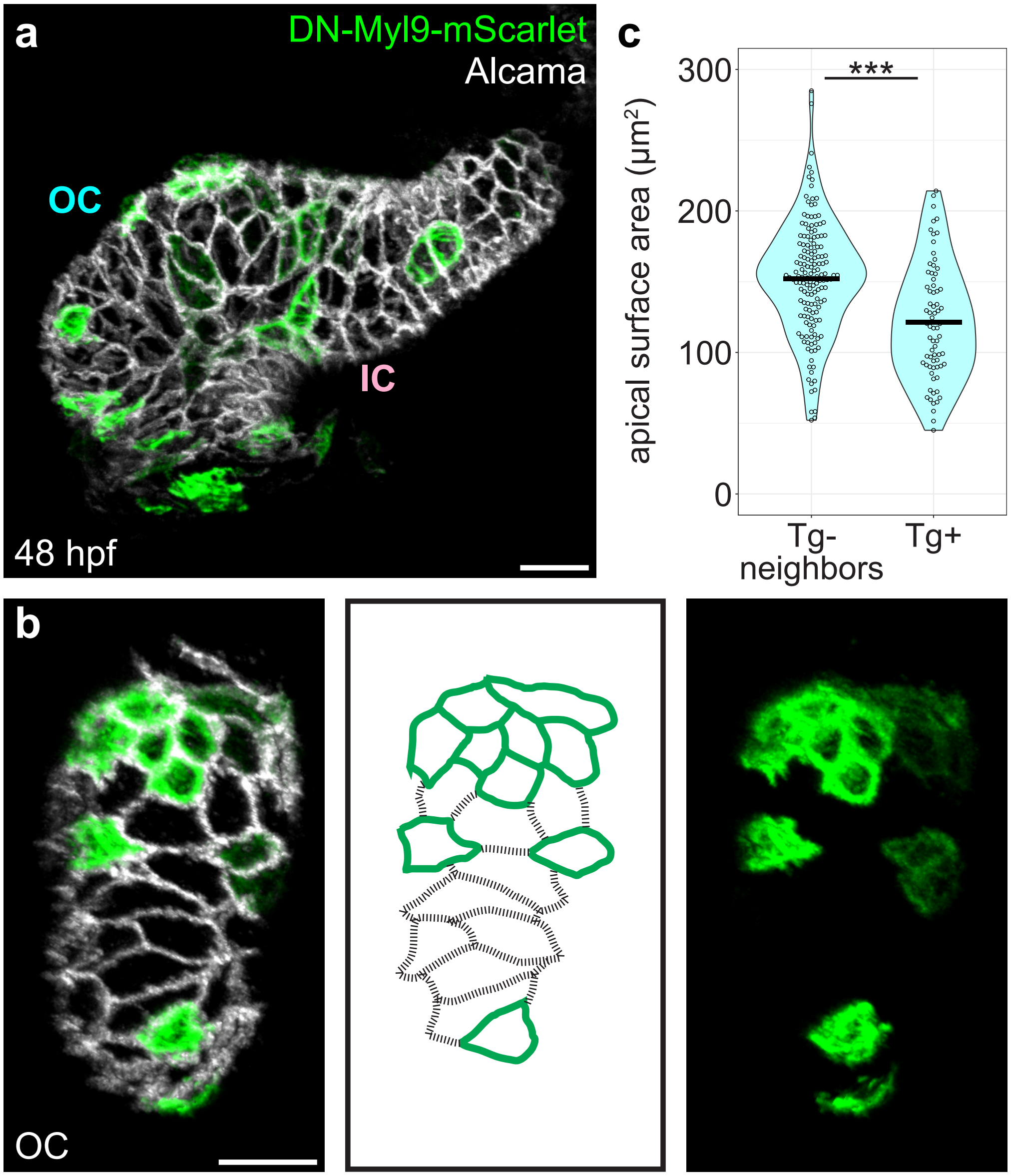

a
DN-Myl9-mScarlet
Alcama
OC
IC
48 hpf
c
300
200
100
0
apical surface area (µm2)
***
Tg-
neighbors
Tg+
b
OC

Supplemental Figure 10. Mosaic expression of injected *Tg(myl7:DN-myl9-mScarlet)* restricts planar expansion in OC cardiomyocytes.

(a) 3D reconstruction of a 48 hpf wild-type heart, following injection with *Tg(myl7:DN-myl9-mScarlet)* and Tol2 mRNA at the one-cell stage, that contains cardiomyocytes with both undetectable (Tg-) and detectable (Tg+) levels of *Tg(myl7:DN-myl9-mScarlet)* expression, based on mScarlet fluorescence. Immunostaining for Alcama labels lateral membranes of cardiomyocytes. **(b)** OC of the heart in (a). Tracings of the cardiomyocytes in (b) are shown to the right. Green outlines indicate Tg+ cardiomyocytes; black outlines indicate Tg- cardiomyocytes. Rightmost panel shows only the green channel, highlighting the variable levels of the DN-myl9-mScarlet fusion protein. **(c)** Violin plot compares apical surface area of Tg- and Tg+ cardiomyocytes. Reduction is comparable to that seen in blastomere transplantation experiments (Fig. 3j). Each dot represents an individual cell. Two-sided Wilcoxon test. (c) $p = 1.91*10^{-7}$. Data gathered from two days of plasmid injection. N=10 embryos. Tg- OC (n=153 cells); Tg+ OC (n=75 cells). Scale bars = 20 μm.

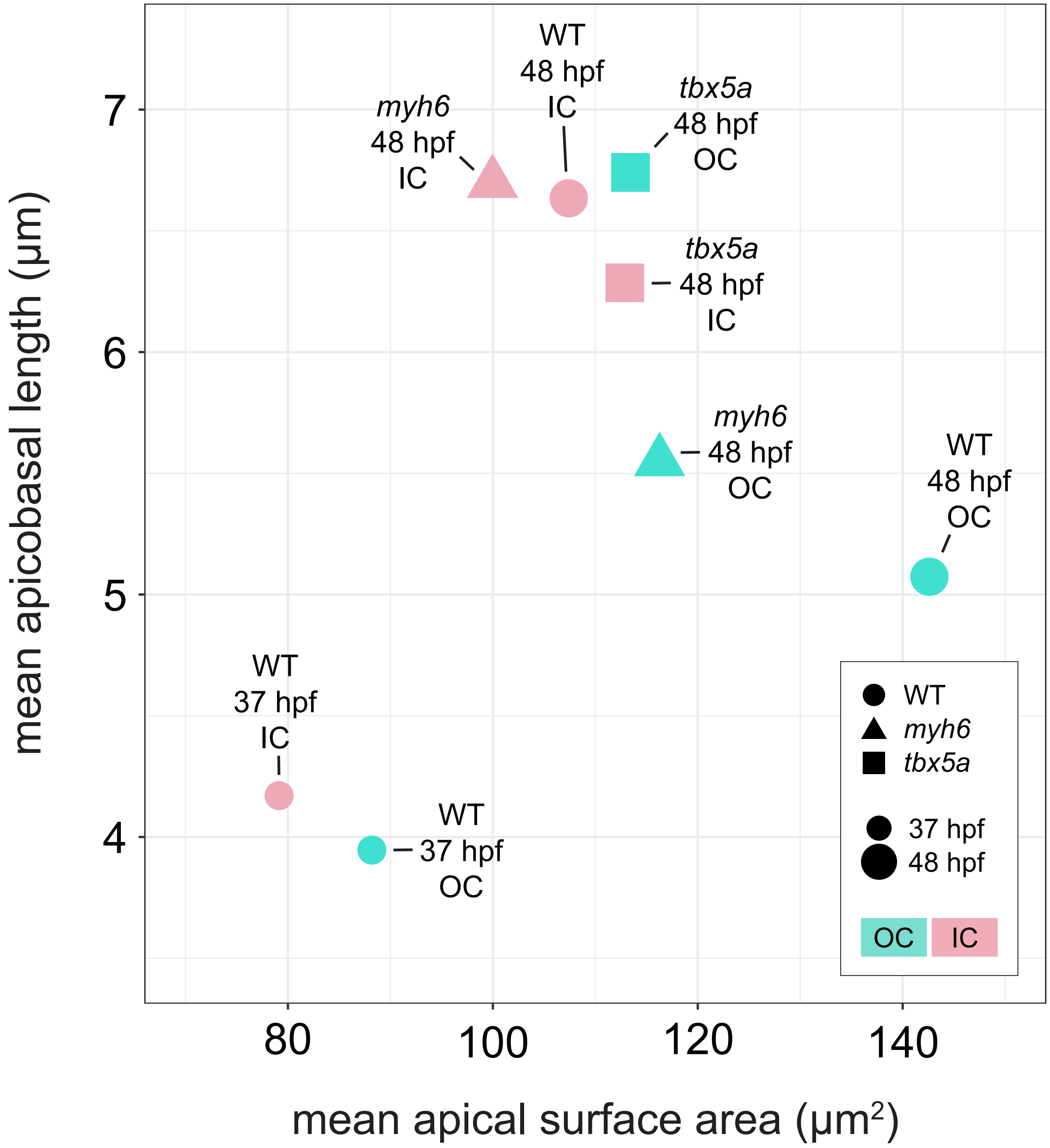

mean apicobasal length (µm)
mean apical surface area (µm²)
7
6
5
4
80
100
120
140
WT 48 hpf IC
myh6 48 hpf IC
tbx5a 48 hpf OC
tbx5a 48 hpf IC
myh6 48 hpf OC
WT 48 hpf OC
WT 37 hpf IC
WT 37 hpf OC
WT
myh6
tbx5a
37 hpf
48 hpf
OC
IC

Supplemental Figure 11. Comparison of the morphologies of OC and IC cardiomyocytes at different developmental stages and with different genotypes.

2D dot plot shows the mean apicobasal length (plotted on the y-axis) and the mean apical surface area (plotted on the x-axis) for ventricular cardiomyocytes in hearts at different developmental stages and with different genotypes. For wild-type, the raw mean values plotted were derived from the data in Fig. 1. For *myh6* and *tbx5a* mutants, the data from Figs. 4 and 6, respectively, were normalized relative to the wild-type 48 hpf data from Fig. 1 (see Methods for more detail on normalization). This visualization highlights several aspects of the relationships between these datasets. For example, wild-type OC and IC cell morphologies are relatively similar at 37 hpf, but they greatly diverge by 48 hpf. Additionally, 48 hpf IC cardiomyocytes of all three genotypes have similar morphologies, whereas OC cardiomyocytes vary based on genotype. The OC cardiomyocytes in *tbx5a* mutants have particularly aberrant morphologies, resembling the IC cardiomyocytes of all three genotypes at 48 hpf.

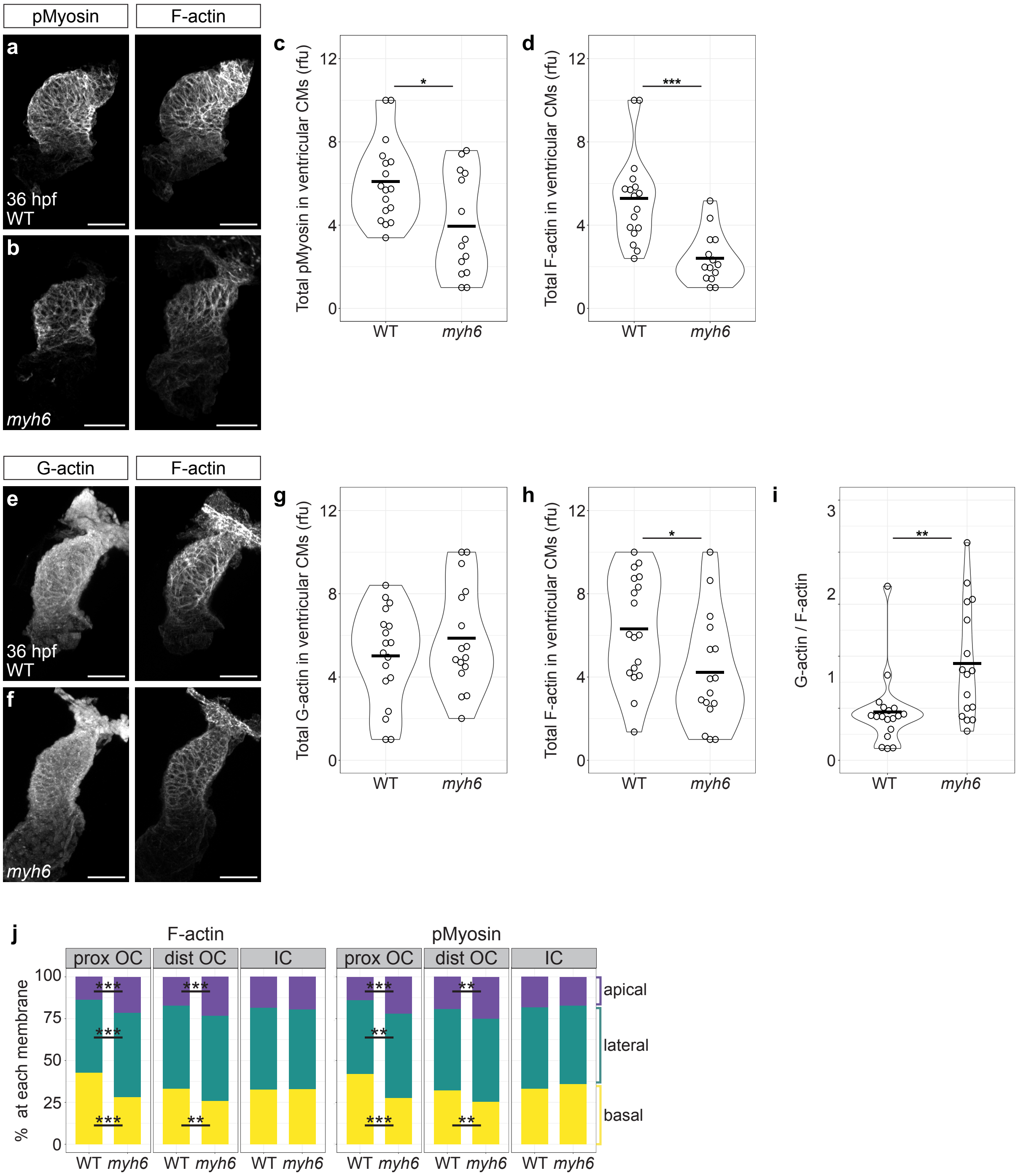
pMyosin
F-actin
a
36 hpf
WT
b
myh6
c
Total pMyosin in ventricular CMs (rfu)
0
4
8
12
*
WT
myh6
d
Total F-actin in ventricular CMs (rfu)
0
4
8
12
***
WT
myh6
G-actin
F-actin
e
36 hpf
WT
f
myh6
g
Total G-actin in ventricular CMs (rfu)
0
4
8
12
WT
myh6
h
Total F-actin in ventricular CMs (rfu)
0
4
8
12
*
WT
myh6
i
G-actin / F-actin
0
1
2
3
**
WT
myh6
j
F-actin
pMyosin
prox OC
dist OC
IC
prox OC
dist OC
IC
% at each membrane
0
25
50
75
100
***
**
apical
lateral
basal
WT myh6
WT myh6
WT myh6
WT myh6
WT myh6
WT myh6

Supplementary Figure 12. Reduced blood flow dampens the total amount of pMyosin and F-actin in the ventricular myocardium.

(**a,b**) 3D reconstructions of a wild-type heart (a) and an *myh6* mutant heart (b) at 36 hpf, immunostained for pMyosin and stained with Phalloidin to label F-actin. (**c,d**) Violin plots compare relative fluorescence units (rfu) of pMyosin (c) and F-actin (d) in the ventricular myocardium between wild-type and mutant embryos. (**e,f**) 3D reconstructions of a wild-type heart (e) and an *myh6* mutant heart (f) at 36 hpf, stained with DNase I and Phalloidin to label G-actin and F-actin, respectively. (**g,h**) Violin plots compare rfu of G-actin (g) and F-actin (h) in the ventricular myocardium between wild-type and mutant embryos. (**i**) Violin plot compares the ratio of G-actin to F-actin between wild-type and mutant embryos. For (c,d,g-i), each dot represents one ventricle, and each black bar represents the mean. Each graph represents two experimental replicates, and each replicate has undergone normalization to a scale of 1-10 (see Methods for more detail). (**j**) Stacked bar charts include the same set of cardiomyocytes as in Fig. 5g,h and show the mean percentage of F-actin or pMyosin at each membrane. Refer to Supplementary Tables 6 and 7 for summary statistics. Two-sided Wilcoxon test. (c) $p = 0.0305$. (d) $p = 0.000108$. (h) $p = 0.0228$. (i) $p = 0.00632$. For F-actin in (j), wild-type proximal OC vs *myh6* proximal OC (basal): $p = 9.48*10^{-9}$; wild-type proximal OC vs *myh6* proximal OC (lateral): $p = 0.000321$; wild-type proximal OC vs *myh6* proximal OC (apical): $p = 4.21*10^{-9}$; wild-type distal OC vs *myh6* distal OC (basal): $p = 0.00172$; wild-type distal OC vs *myh6* distal OC (apical): $p = 4.15*10^{-5}$. For pMyosin in (j), wild-type proximal OC vs *myh6* proximal OC (basal): $p = 5.19*10^{-7}$; wild-type proximal OC vs *myh6* proximal OC (lateral): $p = 0.00717$; wild-type proximal OC vs *myh6* proximal OC (apical): $p = 2.30*10^{-7}$; wild-type distal OC vs *myh6* distal OC (basal): $p = 0.00254$; wild-type distal OC vs *myh6* distal OC (apical): $p = 0.00199$. For (a-d): wild-type (N=17 embryos); *myh6* (N=14 embryos). For (e-i): wild-type (N=18 embryos); *myh6* (N=16 embryos). For (j): wild-type proximal OC (N=5 embryos, n=39 cells); *myh6* proximal OC (N=4 embryos, n=32 cells); wild-type distal OC (N=5 embryos, n=41 cells); *myh6* distal OC (N=4 embryos, n=24 cells); wild-type IC (N=5 embryos, n=54 cells); *myh6* IC (N=4 embryos, n=35 cells). Scale bars = 50 µm.

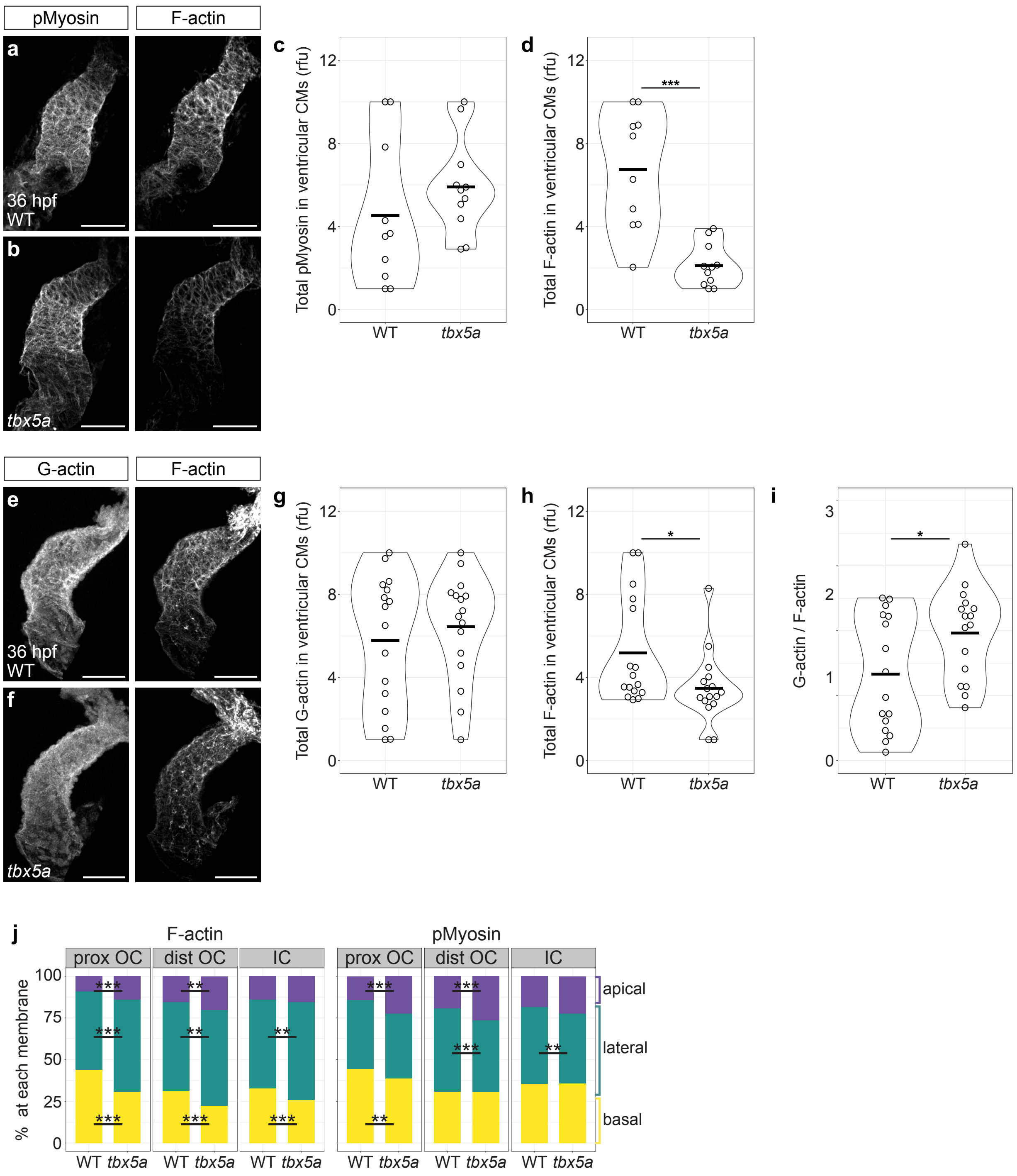

pMyosin
F-actin
a
36 hpf
WT
b
tbx5a
c
Total pMyosin in ventricular CMs (rfu)
0
4
8
12
WT
tbx5a
d
Total F-actin in ventricular CMs (rfu)
***
WT
tbx5a
G-actin
F-actin
e
36 hpf
WT
f
tbx5a
g
Total G-actin in ventricular CMs (rfu)
WT
tbx5a
h
Total F-actin in ventricular CMs (rfu)
*
WT
tbx5a
i
G-actin / F-actin
0
1
2
3
*
WT
tbx5a
j
F-actin
pMyosin
prox OC
dist OC
IC
% at each membrane
0
25
50
75
100
***
**
***
apical
lateral
basal
WT tbx5a

Supplementary Figure 13. ***tbx5a*** **mutants exhibit a reduced amount of total F-actin in the ventricular myocardium.**

(**a,b**) 3D reconstructions of a wild-type heart (a) and a *tbx5a* mutant heart (b) at 36 hpf, immunostained for pMyosin and stained with Phalloidin to label F-actin. (**c,d)** Violin plots compare relative fluorescence units (rfu) of pMyosin (c) and F-actin (d) in the ventricular myocardium between wild-type and mutant embryos. (**e,f**) 3D reconstructions of a wild-type heart (e) and a *tbx5a* mutant heart (f) at 36 hpf, stained with DNase I and Phalloidin to label G-actin and F-actin, respectively. (**g,h**) Violin plots compare rfu of G-actin (g) and F-actin (h) in the ventricular myocardium between wild-type and mutant embryos. (**i**) Violin plot compares the ratio of G-actin to F-actin between wild-type and mutant embryos. For (c,d,g-i), each dot represents one ventricle, and each black bar represents the mean. Each graph represents two experimental replicates, and each replicate has undergone normalization to a scale of 1-10 (see Methods for more detail). (**j**) Stacked bar charts include the same set of cardiomyocytes as in Fig. 7g,h and show the mean percentage of F-actin or pMyosin at each membrane. Refer to Supplementary Tables 8 and 9 for summary statistics. Two-sided Wilcoxon test. (d) $p = 0.000632$. (h) $p = 0.0437$. (i) $p = 0.0468$. For F-actin in (j), wild-type proximal OC vs *tbx5a* proximal OC (basal): $p = 5.81*10^{-10}$; wild-type proximal OC vs *tbx5a* proximal OC (lateral): $p = 1.33*10^{-5}$; wild-type proximal OC vs *tbx5a* proximal OC (apical): $p = 0.000230$; wild-type distal OC vs *tbx5a* distal OC (basal): $p = 9.57*10^{-7}$; wild-type distal OC vs *tbx5a* distal OC (lateral): $p = 0.00322$; wild-type distal OC vs *tbx5a* distal OC (apical): $p = 0.00813$; wild-type IC vs *tbx5a* IC (basal): $p = 6.10*10^{-5}$; wild-type IC vs *tbx5a* IC (lateral): $p = 0.00216$. For pMyosin in (j), wild-type proximal OC vs *tbx5a* proximal OC (basal): $p = 0.00958$; wild-type proximal OC vs *tbx5a* proximal OC (apical): $p = 9.92*10^{-7}$; wild-type distal OC vs *tbx5a* distal OC (lateral): $p = 4.22*10^{-5}$; wild-type distal OC vs *tbx5a* distal OC (apical): $p = 0.000217$; wild-type IC vs *tbx5a* IC (lateral): $p = 0.00139$. For (a-d): wild-type (N=10 embryos); *tbx5a* (N=11 embryos). For (e-i): wild-type (N=16 embryos); *myh6* (N=16 embryos). For (j): wild-type proximal OC (N=5 embryos, n=58 cells); *tbx5a* proximal OC (N=5 embryos, n=64 cells); wild-type distal OC (N=5 embryos, n=61 cells); *tbx5a* distal OC (N=5 embryos, n=67 cells); wild-type IC (N=5 embryos, n=69 cells); *tbx5a* IC (N=5 embryos, n=81 cells). Scale bars = 50 µm.

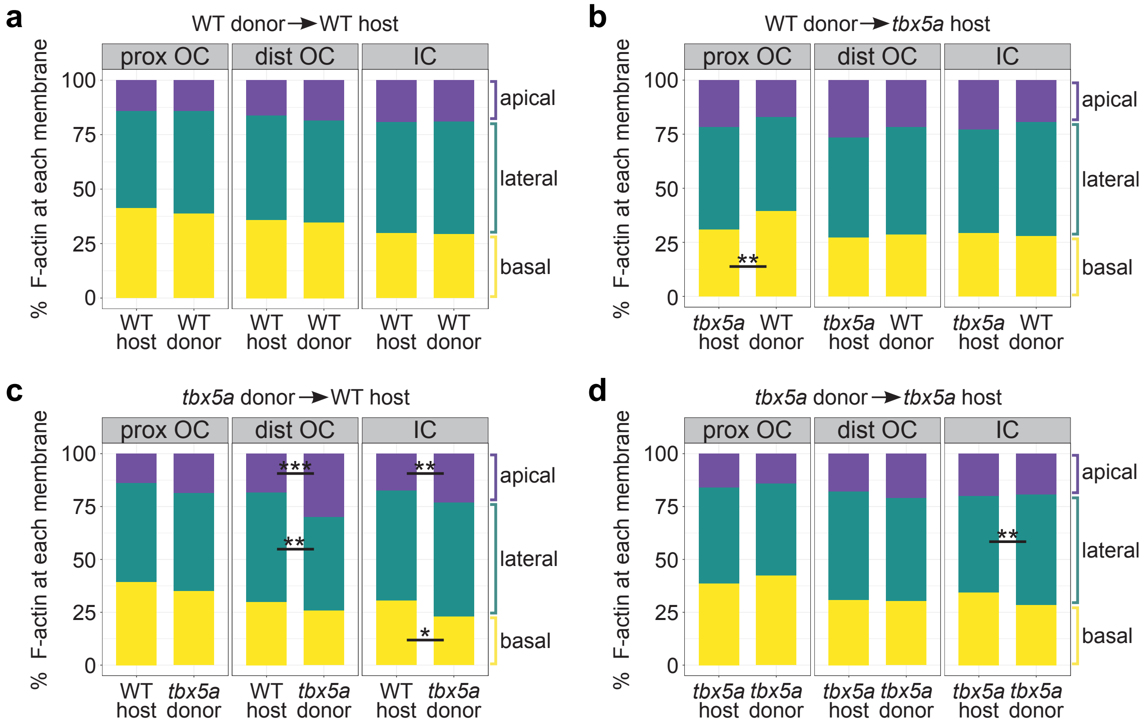

a
WT donor → WT host
prox OC
dist OC
IC
% F-actin at each membrane
100
75
50
25
0
apical
lateral
basal
WT host
WT donor
b
WT donor → tbx5a host
tbx5a host
WT donor
**
c
tbx5a donor → WT host
WT host
tbx5a donor
***
**
*
d
tbx5a donor → tbx5a host
tbx5a host
tbx5a donor
**

Supplementary Figure 14. Subcellular F-actin organization in mosaic hearts from Fig. 8, shown as percentage of F-actin at each membrane.

(**a-d**) Stacked bar charts include the same sets of cardiomyocytes as in Fig. 8g,j,m,p and show the mean percentage of F-actin at each membrane. Refer to Supplementary Tables 10, 11, 12, and 13 for summary statistics. Note that, in the *tbx5a* mutant into wild-type scenario (c), some F-actin proportions are significantly different between host- and donor-derived cardiomyocytes in the distal OC and the IC. However, the nature of these differences do not translate into differences between the ratios of (basal F-actin / (apical + lateral F-actin)) presented in Fig. 8m. Two-sided Wilcoxon test. (b) $p = 0.00524$. (c) wild-type host distal OC vs *tbx5a* donor distal OC (lateral): $p = 0.00389$; wild-type host distal OC vs *tbx5a* donor distal OC (apical): $p = 0.000671$; wild-type host IC vs *tbx5a* donor IC (basal): $p = 0.0221$; wild-type host IC vs *tbx5a* donor IC (apical): $p = 0.00378$. (d) $p = 0.00811$. Data gathered from eight days of transplantation. For WT into WT transplants: host proximal OC (N=6 embryos, n=55 cells); donor proximal OC (N=7 embryos, n=14 cells); host distal OC (N=5 embryos, n=30 cells); donor distal OC (N=6 embryos, n=29 cells); host IC (N=4 embryos, n=30 cells); donor IC (N=4 embryos, n=29 cells). For WT into *tbx5a* transplants: host proximal OC (N=5 embryos, n=40 cells); donor proximal OC (N=5 embryos, n=25 cells); host distal OC (N=5 embryos, n=40 cells); donor distal OC (N=5 embryos, n=35 cells); host IC (N=5 embryos, n=18 cells); donor IC (N=5 embryos, n=14 cells). For *tbx5a* into WT transplants: host proximal OC (N=7 embryos, n=38 cells); donor proximal OC (N=5 embryos, n=14 cells); host distal OC (N=6 embryos, n=28 cells); donor distal OC (N=5 embryos, n=13 cells); host IC (N=5 embryos, n=44 cells); donor IC (N=5 embryos, n=18 cells). For *tbx5a* into *tbx5a* transplants: host proximal OC (N=4 embryos, n=16 cells); donor proximal OC (N=2 embryos, n=3 cells); host distal OC (N=4 embryos, n=21 cells); donor distal OC (N=4 embryos, n=14 cells); host IC (N=5 embryos, n=36 cells); donor IC (N=5 embryos, n=14 cells).

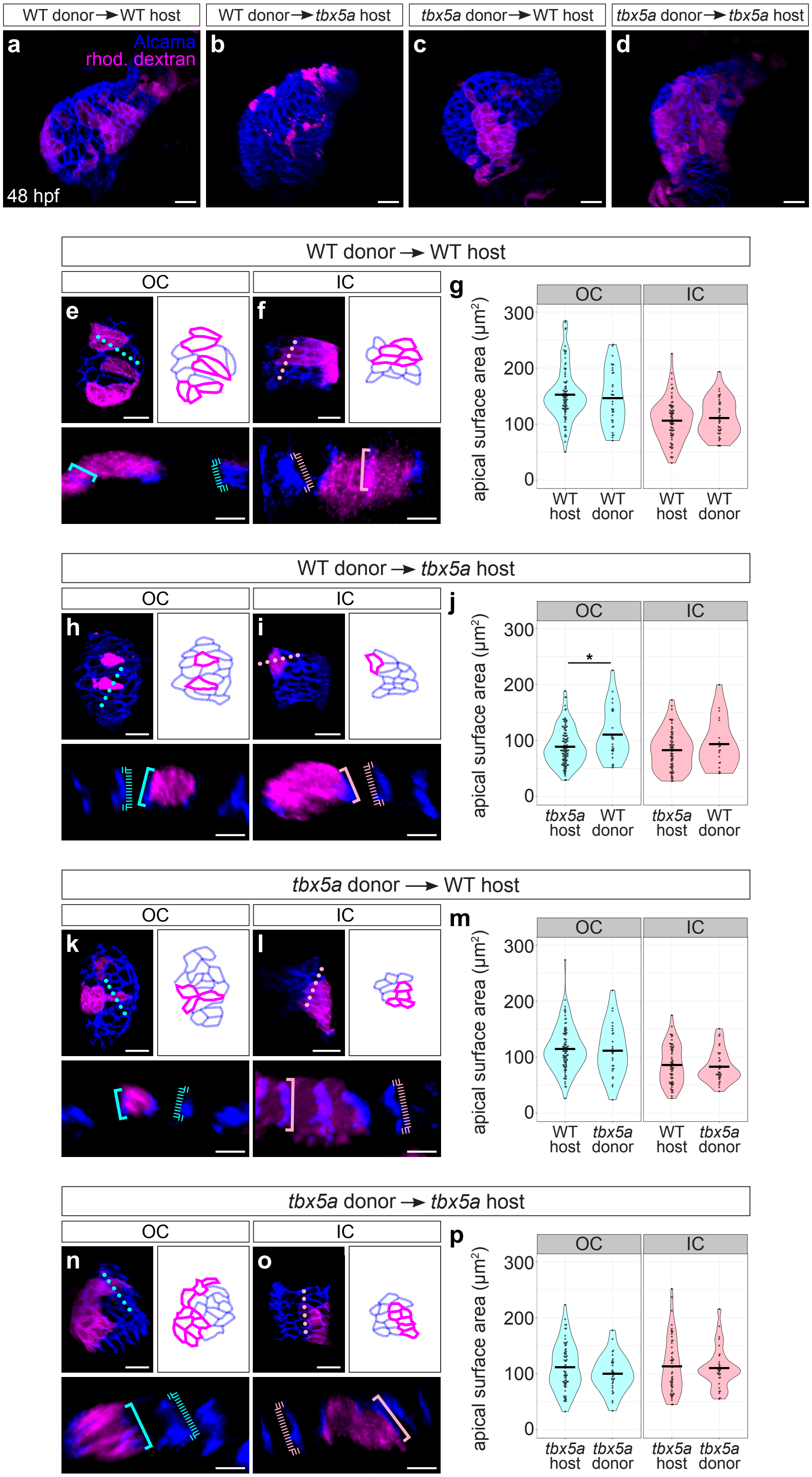
WT donor → WT host
WT donor → tbx5a host
tbx5a donor → WT host
tbx5a donor → tbx5a host
a
Alcama
rhod. dextran
48 hpf
b
c
d
WT donor → WT host
OC
IC
e
f
g
apical surface area (μm²)
300
200
100
0
WT host
WT donor
WT donor → tbx5a host
h
i
j
*
tbx5a host
tbx5a donor → WT host
k
l
m
tbx5a donor
tbx5a donor → tbx5a host
n
o
p

Supplementary Figure 15. *tbx5a* functions in a partially cell-autonomous manner to support planar expansion of OC cardiomyocytes.

(**a-d**) 3D reconstructions show examples of mosaic 48 hpf hearts resulting from blastomere transplantation. Immunostaining for Alcama labels lateral membranes of cardiomyocytes (blue), and donor-derived cells are labeled with rhodamine dextran (magenta). OCs (**e,h,k,n**) and ICs (**f,i,l,o**) show additional examples of each transplant scenario. Tracing of cells in (e,f,h,i,k,l,n,o) use magenta outlines to indicate donor-derived cardiomyocytes and blue outlines to indicate host-derived cells. Cross-sections in (e,f,h,i,k,l,n,o) are through positions indicated by dotted lines; blue and pink brackets highlight apicobasal length of individual cells, with dashed brackets for host-derived cells and solid brackets for donor-derived cardiomyocytes. (**g,j,m,p**) Violin plots compare apical surface area of host-derived cells to those of donor-derived cardiomyocytes. Each dot represents an individual cell. Two-sided Wilcoxon test. (j) $p = 0.0319$. Data gathered from ten days of transplantation. For WT into WT transplants: host OC (N=7 embryos, n=92 cells); donor OC (N=7 embryos, n=34 cells); host IC (N=6 embryos, n=73 cells); donor IC (N=6 embryos, n=39 cells). For WT into *tbx5a* transplants: host OC (N=7 embryos, n=123 cells); donor OC (N=7 embryos, n=27 cells); host IC (N=7 embryos, n=72 cells); donor IC (N=7 embryos, n=18 cells). For *tbx5a* into WT transplants: host OC (N=8 embryos, n=93 cells); donor OC (N=8 embryos, n=28 cells); host IC (N=5 embryos, n=59 cells); donor IC (N=5 embryos, n=34 cells). For *tbx5a* into *tbx5a* transplants: host OC (N=3 embryos, n=67 cells); donor OC (N=3 embryos, n=32 cells); host IC (N=3 embryos, n=56 cells); donor IC (N=3 embryos, n=30 cells). Scale bars = 20 µm in (a-d) and in views of OCs; 5 µm in cross-sections.

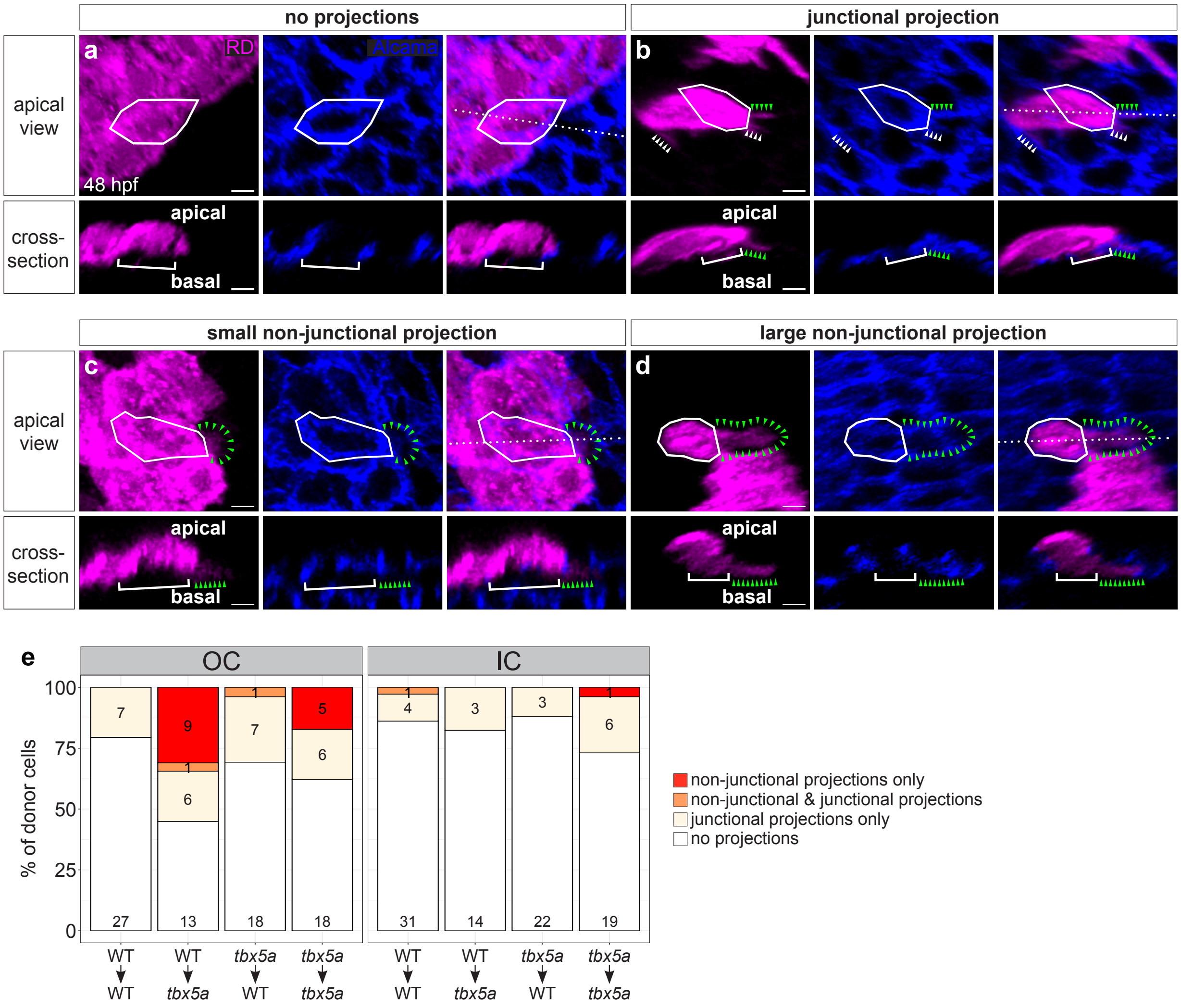

no projections
junctional projection
apical view
cross-section
a
RD
48 hpf
apical
basal
b
small non-junctional projection
large non-junctional projection
c
d
e
OC
IC
% of donor cells
100
75
50
25
0
7
27
9
1
6
13
1
7
18
5
6
18
1
4
31
3
14
3
22
1
6
19
WT
tbx5a
non-junctional projections only
non-junctional & junctional projections
junctional projections only
no projections

Supplementary Figure 16. Wild-type cardiomyocytes extend excessive basal projections when positioned next to *tbx5a* mutant cardiomyocytes.

(**a-d**) Apical views of mosaic hearts at 48 hpf provide examples of donor cardiomyocytes that can be categorized by their types of basal projections. Immunostaining for Alcama labels lateral membranes of cardiomyocytes (blue), and donor-derived cardiomyocytes are labeled with rhodamine dextran (RD, magenta). In each panel, the apical surface of a single donor-derived cardiomyocyte is outlined. Cross-sections shown are through outlined cardiomyocytes at the positions noted by dotted lines. Solid brackets highlight the width of the main mass of the outlined donor-derived cardiomyocyte; green arrowheads highlight the basal projection. Cells with projections were detected in both proximal and distal regions of the curvatures. (a) Outlined cardiomyocyte has no visible projections. (b) Outlined cardiomyocyte has only thin projections (arrowheads) that extend along the junction between two neighboring cells. (c) Outlined cardiomyocyte has one small non-junctional projection (a broader projection that extends underneath a neighboring cell, instead of between two neighbors), outlined with arrowheads. (d) Outlined cardiomyocyte has one large non-junctional projection, outlined with arrowheads. (**e**) Stacked bar charts show the proportions of donor-derived cardiomyocytes exhibiting different categories of projections, organized by transplant scenario and curvature. Only donor-derived cardiomyocytes that directly contact host-derived cardiomyocytes were considered in this analysis. Numerals on the charts refer to the number of donor cells in each category. Scale bars = 5 μm.

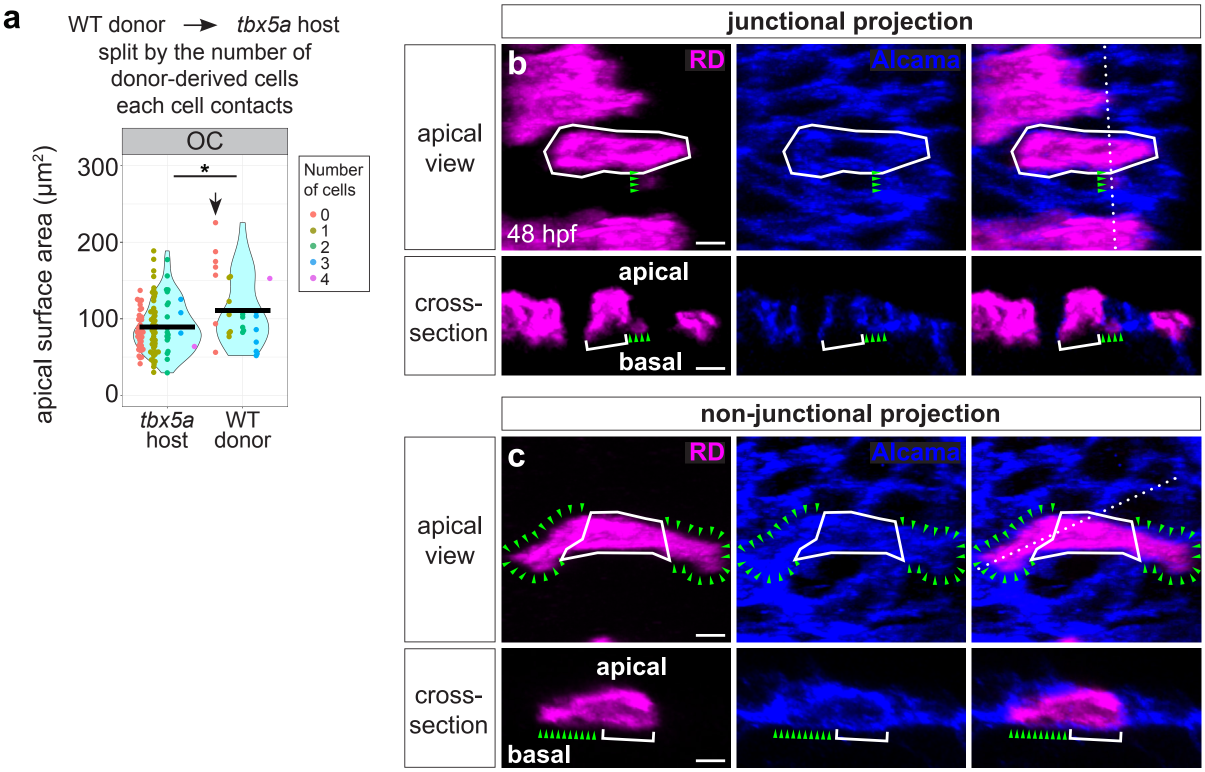
a
WT donor → tbx5a host
split by the number of
donor-derived cells
each cell contacts
OC
apical surface area (µm²)
300
200
100
0
*
Number of cells
0
1
2
3
4
tbx5a host
WT donor
junctional projection
apical view
cross-section
b
RD
Alcama
48 hpf
apical
basal
non-junctional projection
c
RD
Alcama
apical
basal

Supplementary Figure 17. Single-cell wild-type clones in a *tbx5a* mutant OC send basal projections between and under neighboring host-derived cardiomyocytes.

(**a**) OC data from Supplementary Fig. 15j, with individual data points colored based on how many other donor-derived cardiomyocytes that cardiomyocyte contacts. A donor-derived cardiomyocyte in contact with no other donor-derived cardiomyocytes is considered a single-cell clone (coral dots; arrow). These 7 cardiomyocytes have, on average, a larger apical surface area than donor-derived cardiomyocytes in contact with other donor-derived cardiomyocytes. Two-sided Wilcoxon test compares host and donor values. (a) $p = 0.0319$. (**b,c**) Apical views of two examples of the 7 donor-derived cardiomyocytes highlighted in (a). Immunostaining for Alcama labels lateral membranes of cardiomyocytes (blue), and donor-derived cells are labeled with rhodamine dextran (RD, magenta). In each panel, the apical surface of a single donor-derived cardiomyocyte is outlined with a solid line. Cross-sections are through outlined cardiomyocytes at the positions noted by dotted lines; solid brackets highlight the width of the main mass of the outlined donor-derived cardiomyocyte. (b) Three of the 7 single-cell donor-derived clones highlighted in (a) send thin basal projections that extend along the junction between two neighboring host-derived cardiomyocytes, as in this example with one such projection denoted with green arrowheads. (c) The other 4 of the 7 single-cell donor-derived clones highlighted in (a) send broader basal projections underneath neighboring host-derived cardiomyocytes, as in this example with two broad non-junctional projections outlined with green arrowheads. Scale bars = 5 μm.

Genotype/Stage	Location	# of cells	Membrane	Mean %	S.D.	S.E.M.	Range of %
wild-type 36 hpf	OC	199	apical	13.7	6.5	0.5	2.0 - 41.7
			basal	37.6	11.2	0.8	11.8 - 66.4
			lateral	48.7	8.8	0.6	19.4 - 71.3
	IC	123	apical	16.0	5.9	0.5	3.0 - 32.0
			basal	32.8	9.3	0.8	15.7 - 56.2
			lateral	51.2	8.6	0.8	25.6 - 69.7

Supplementary Table 1. Summary statistics corresponding to F-actin localization in Fig. 2e. The stacked bar chart in Fig. 2e shows the mean percentage of F-actin at each membrane. These summary statistics provide information for assessing the distribution of these data. S.D.: standard deviation; S.E.M.: standard error of the mean.

Genotype/Stage	Location	# of cells	Membrane	Mean %	S.D.	S.E.M.	Range of %
wild-type 36 hpf	OC	174	apical	16.7	6.9	0.5	4.8 - 43.7
			basal	37.3	12.0	0.9	10.2 - 65.6
			lateral	45.9	8.6	0.6	23.3 - 67.8
	IC	109	apical	18.4	6.7	0.6	5.6 - 39.4
			basal	34.4	10.7	1.0	16.7 - 59.5
			lateral	47.3	7.8	0.8	24.3 - 68.5

Supplementary Table 2. Summary statistics corresponding to pMyosin localization in Fig. 2e. Information is presented as in Supplementary Table 1.

Genotype/Stage	Location	# of cells	Membrane	Mean %	S.D.	S.E.M.	Range of %
wild-type 36 hpf	proximal OC	97	apical	10.9	5.1	0.5	2.0 - 24.6
			basal	43.5	9.6	1.0	22.2 - 66.4
			lateral	45.6	8.2	0.8	28.4 - 64.5
	distal OC	102	apical	16.3	6.6	0.7	4.7 - 41.7
			basal	32.0	9.7	1.0	11.8 - 60.0
			lateral	51.6	8.2	0.8	19.4 - 71.3
	proximal IC	63	apical	15.1	5.8	0.7	3.0 - 29.9
			basal	34.7	9.0	1.1	16.1 - 56.2
			lateral	50.2	8.5	1.1	25.6 - 69.7
	distal IC	60	apical	16.9	5.9	0.8	5.7 - 32.0
			basal	30.9	9.2	1.2	15.7 - 53.8
			lateral	52.2	8.5	1.1	34.1 - 68.8

Supplementary Table 3. Summary statistics corresponding to F-actin localization in Supplementary Fig. 4d. Information is presented as in Supplementary Table 1.

Genotype/Stage	Location	# of cells	Membrane	Mean %	S.D.	S.E.M.	Range of %
wild-type 36 hpf	proximal OC	85	apical	14.1	5.3	0.6	4.8 - 28.7
			basal	43.4	11.0	1.2	19.6 - 65.6
			lateral	42.5	8.2	0.9	25.7 - 62.0
	distal OC	89	apical	19.2	7.4	0.8	8.7 - 43.7
			basal	31.6	9.9	1.0	10.2 - 52.6
			lateral	49.2	7.6	0.8	23.3 - 67.8
	proximal IC	56	apical	18.0	6.6	0.9	5.6 - 39.4
			basal	36.0	10.6	1.4	17.2 - 59.5
			lateral	46.0	7.8	1.0	24.3 - 61.4
	distal IC	53	apical	18.8	7.0	1.0	8.1 - 33.4
			basal	32.6	10.6	1.5	16.7 - 54.1
			lateral	48.6	7.8	1.1	35.2 - 68.6

Supplementary Table 4. Summary statistics corresponding to pMyosin localization in Supplementary Fig. 4d. Information is presented as in Supplementary Table 1.

Location	Genotype/Stage	# of cells	Membrane	Mean %	S.D.	S.E.M.	Range of %
proximal OC	*WT myl9* 36 hpf	39	apical	11.3	3.1	0.5	7.1 - 19.1
			basal	44.4	10.6	1.7	22.9 - 62.2
			lateral	44.4	8.7	1.4	28.3 - 63.3
	DN myl9 36 hpf	45	apical	14.6	7.3	1.1	5.2 - 42.2
			basal	35.7	12.3	1.8	14.8 - 68.8
			lateral	49.7	12.0	1.8	15.4 - 80.0
distal OC	*WT myl9* 36 hpf	42	apical	15.1	5.0	0.8	7.6 - 25.7
			basal	37.5	10.9	1.7	16.2 - 57.1
			lateral	47.4	8.3	1.3	24.7 - 67.5
	DN myl9 36 hpf	46	apical	16.1	4.7	0.7	6.8 - 26.7
			basal	33.8	11.6	1.7	17.8 - 76.0
			lateral	50.1	9.1	1.3	17.2 - 67.5
IC	*WT myl9* 36 hpf	50	apical	13.2	3.6	0.5	7.5 - 22.3
			basal	37.8	10.0	1.4	16.5 - 55.8
			lateral	48.9	7.9	1.1	34.9 - 65.4
	DN myl9 36 hpf	53	apical	14.2	5.7	0.8	4.7 - 36.7
			basal	35.8	9.1	1.3	17.4 - 53.7
			lateral	50.0	7.8	1.1	36.5 - 67.6

Supplementary Table 5. Summary statistics corresponding to F-actin localization in Supplementary Fig. 9e. Information is presented as in Supplementary Table 1.

Location	Genotype/Stage	# of cells	Membrane	Mean %	S.D.	S.E.M.	Range of %
proximal OC	wild-type 36 hpf	39	apical	13.7	4.9	0.8	6.0 - 24.6
			basal	42.8	9.5	1.5	22.5 - 63.8
			lateral	43.6	8.1	1.3	28.4 - 61.6
	myh6 36 hpf	32	apical	21.6	4.6	0.8	14.3 - 32.3
			basal	28.1	8.3	1.5	12.1 - 42.7
			lateral	50.3	7.6	1.3	38.1 - 69.0
distal OC	wild-type 36 hpf	41	apical	17.3	6.0	0.9	8.9 - 33.5
			basal	33.2	9.7	1.5	11.8 - 52.7
			lateral	49.5	8.8	1.4	19.4 - 66.0
	myh6 36 hpf	24	apical	23.3	6.1	1.2	16.4 - 41.6
			basal	25.9	5.6	1.1	16.6 - 35.4
			lateral	50.8	5.8	1.2	39.5 - 61.9
IC	wild-type 36 hpf	54	apical	18.5	4.8	0.7	9.7 - 29.9
			basal	32.7	8.6	1.2	17.5 - 54.3
			lateral	48.7	7.4	1.0	26.6 - 62.5
	myh6 36 hpf	35	apical	19.7	4.6	0.8	10.0 - 29.0
			basal	32.9	10.4	1.8	10.5 - 54.7
			lateral	47.5	7.6	1.3	31.8 - 65.9

Supplementary Table 6. Summary statistics corresponding to F-actin localization in Supplementary Fig. 12j. Information is presented as in Supplementary Table 1.

Location	Genotype/Stage	# of cells	Membrane	Mean %	S.D.	S.E.M.	Range of %
proximal OC	wild-type 36 hpf	39	apical	13.8	5.9	0.9	4.8 - 28.1
			basal	42.1	10.5	1.7	23.6 - 60.4
			lateral	44.1	7.8	1.3	33.0 - 62.0
	myh6 36 hpf	32	apical	22.2	6.2	1.1	9.9 - 35.1
			basal	27.6	11.0	1.9	9.6 - 53.6
			lateral	50.2	9.3	1.6	36.3 - 72.9
distal OC	wild-type 36 hpf	41	apical	19.2	7.6	1.2	8.7 - 36.9
			basal	32.3	9.0	1.4	10.2 - 49.0
			lateral	48.5	7.3	1.1	23.3 - 60.9
	myh6 36 hpf	24	apical	25.2	7.0	1.4	15.6 - 41.1
			basal	25.4	8.6	1.8	11.2 - 45.4
			lateral	49.5	5.8	1.2	34.8 - 61.4
IC	wild-type 36 hpf	54	apical	18.2	6.9	0.9	9.1 - 39.4
			basal	33.2	10.3	1.4	17.1 - 54.1
			lateral	48.6	7.2	1.0	29.4 - 68.5
	myh6 36 hpf	35	apical	17.3	4.9	0.8	6.3 - 28.0
			basal	35.9	12.1	2.0	11.0 - 61.1
			lateral	46.8	8.8	1.5	31.3 - 66.4

Supplementary Table 7. Summary statistics corresponding to pMyosin localization in Supplementary Fig. 12j. Information is presented as in Supplementary Table 1.

Location	Genotype/Stage	# of cells	Membrane	Mean %	S.D.	S.E.M.	Range of %
proximal OC	wild-type 36 hpf	58	apical	9.0	4.4	0.6	2.0 - 22.3
			basal	44.0	9.7	1.3	22.2 - 66.4
			lateral	47.0	8.1	1.1	29.9 - 64.5
	tbx5a 36 hpf	64	apical	14.0	8.2	1.0	3.6 - 42.3
			basal	30.8	10.1	1.3	11.1 - 55.2
			lateral	55.2	10.6	1.3	29.0 - 82.3
distal OC	wild-type 36 hpf	61	apical	15.7	7.0	0.9	4.7 - 41.7
			basal	31.2	9.8	1.2	13.7 - 60.0
			lateral	53.1	7.6	1.0	32.8 - 71.3
	tbx5a 36 hpf	67	apical	20.4	10.1	1.2	5.7 - 55.2
			basal	22.2	9.1	1.1	5.7 - 45.1
			lateral	57.4	9.5	1.2	36.8 - 76.9
IC	wild-type 36 hpf	69	apical	14.0	5.9	0.7	3.0 - 32.0
			basal	32.8	9.8	1.2	15.7 - 56.2
			lateral	53.1	9.0	1.1	25.6 - 69.7
	tbx5a 36 hpf	81	apical	15.6	9.4	1.1	3.2 - 38.9
			basal	25.9	10.3	1.2	5.9 - 50.9
			lateral	58.6	9.3	1.0	41.9 - 81.0

Supplementary Table 8. Summary statistics corresponding to F-actin localization in Supplementary Fig. 13j. Information is presented as in Supplementary Table 1.

Location	Genotype/Stage	# of cells	Membrane	Mean %	S.D.	S.E.M.	Range of %
proximal OC	wild-type 36 hpf	46	apical	14.4	4.7	0.7	8.3 - 28.7
			basal	44.5	11.4	1.7	19.6 - 65.6
			lateral	41.1	8.4	1.2	25.7 - 60.4
	tbx5a 36 hpf	64	apical	22.5	9.3	1.2	8.0 - 49.2
			basal	38.9	10.9	1.4	16.6 - 64.4
			lateral	38.6	7.4	0.9	20.1 - 59.9
distal OC	wild-type 36 hpf	48	apical	19.2	7.2	1.0	9.4 - 43.7
			basal	30.9	10.6	1.5	14.4 - 52.6
			lateral	49.9	7.7	1.1	35.5 - 67.8
	tbx5a 36 hpf	67	apical	26.5	11.0	1.4	9.0 - 54.6
			basal	30.5	11.1	1.4	8.4 - 53.1
			lateral	43.1	7.6	0.9	24.4 - 64.7
IC	wild-type 36 hpf	55	apical	18.5	6.7	0.9	5.6 - 33.4
			basal	35.5	11.1	1.5	16.7 - 59.5
			lateral	46.0	8.3	1.1	24.3 - 66.0
	tbx5a 36 hpf	81	apical	22.6	11.0	1.2	7.5 - 49.5
			basal	35.8	11.6	1.3	10.9 - 57.8
			lateral	41.6	7.2	0.8	27.3 - 58.5

Supplementary Table 9. Summary statistics corresponding to pMyosin localization in Supplementary Fig. 13j. Information is presented as in Supplementary Table 1.

Location	Type of cell/Stage	# of cells	Membrane	Mean %	S.D.	S.E.M.	Range of %
proximal OC	wild-type host 36 hpf	55	apical	14.2	5.3	0.7	4.2 - 28.8
			basal	41.4	10.8	1.5	21.9 - 63.9
			lateral	44.3	7.5	1.0	30.9 - 63.2
	wild-type donor 36 hpf	14	apical	14.2	5.0	1.3	7.2 - 25.6
			basal	38.9	8.0	2.1	24.6 - 50.5
			lateral	46.9	7.0	1.9	38.9 - 62.9
distal OC	wild-type host 36 hpf	30	apical	16.3	4.5	0.8	6.5 - 24.0
			basal	35.8	7.5	1.4	19.2 - 46.4
			lateral	47.9	8.1	1.5	31.0 - 66.6
	wild-type donor 36 hpf	29	apical	18.6	6.5	1.2	11.1 - 40.0
			basal	34.7	10.5	1.9	13.9 - 50.7
			lateral	46.7	6.5	1.2	34.3 - 58.8
IC	wild-type host 36 hpf	30	apical	19.1	5.5	1.0	7.0 - 31.5
			basal	30.0	11.1	2.0	10.1 - 51.5
			lateral	50.8	9.2	1.7	34.6 - 69.6
	wild-type donor 36 hpf	29	apical	19.1	4.0	0.7	12.2 - 27.6
			basal	29.4	8.7	1.6	15.5 - 45.0
			lateral	51.5	8.0	1.5	34.3 - 64.2

Supplementary Table 10. Summary statistics corresponding to F-actin localization in Supplementary Fig. 14a. Information is presented as in Supplementary Table 1.

Location	Type of cell/Stage	# of cells	Membrane	Mean %	S.D.	S.E.M.	Range of %
proximal OC	*tbx5a* host 36 hpf	40	apical	21.7	10.5	1.7	3.5 - 51.1
			basal	31.0	12.0	1.9	8.8 - 60.1
			lateral	47.3	7.3	1.2	32.4 - 61.3
	wild-type donor 36 hpf	25	apical	17.2	6.5	1.3	7.6 - 33.8
			basal	39.4	11.7	2.3	15.4 - 59.0
			lateral	43.3	6.8	1.4	30.2 - 55.4
distal OC	*tbx5a* host 36 hpf	40	apical	26.5	10.3	1.6	9.7 - 53.4
			basal	27.4	8.6	1.4	11.8 - 45.5
			lateral	46.1	8.6	1.4	25.8 - 62.8
	wild-type donor 36 hpf	35	apical	21.7	7.7	1.3	5.7 - 39.7
			basal	28.7	7.6	1.3	18.4 - 49.1
			lateral	49.6	9.4	1.6	23.1 - 67.7
IC	*tbx5a* host 36 hpf	18	apical	22.8	6.0	1.4	12.2 - 36.9
			basal	29.4	8.2	1.9	15.2 - 41.4
			lateral	47.8	9.4	2.2	32.6 - 72.6
	wild-type donor 36 hpf	14	apical	19.5	4.7	1.3	10.9 - 29.7
			basal	27.9	6.5	1.7	18.1 - 44.4
			lateral	52.6	6.3	1.7	41.2 - 66.8

Supplementary Table 11. Summary statistics corresponding to F-actin localization in Supplementary Fig. 14b. Information is presented as in Supplementary Table 1.

Location	Type of cell/Stage	# of cells	Membrane	Mean %	S.D.	S.E.M.	Range of %
proximal OC	wild-type host 36 hpf	38	apical	14.0	5.7	0.9	5.0 - 27.3
			basal	39.3	10.3	1.7	12.6 - 58.6
			lateral	46.7	7.7	1.2	28.4 - 61.6
	tbx5a donor 36 hpf	14	apical	18.6	10.5	2.8	5.4 - 41.1
			basal	35.1	11.2	3.0	16.9 - 59.3
			lateral	46.3	7.5	2.0	33.1 - 58.6
distal OC	wild-type host 36 hpf	28	apical	18.5	8.6	1.6	6.8 - 43.5
			basal	29.9	10.2	1.9	10.3 - 45.7
			lateral	51.6	6.0	1.1	43.2 - 65.1
	tbx5a donor 36 hpf	13	apical	30.0	10.0	2.8	8.9 - 43.8
			basal	26.0	6.7	1.9	15.2 - 33.6
			lateral	44.0	8.9	2.5	32.5 - 61.9
IC	wild-type host 36 hpf	44	apical	17.3	6.3	0.9	8.6 - 37.7
			basal	30.7	12.0	1.8	12.1 - 56.7
			lateral	52.0	9.8	1.5	31.1 - 73.3
	tbx5a donor 36 hpf	18	apical	23.0	7.6	1.8	9.3 - 39.1
			basal	23.1	9.4	2.2	9.8 - 41.0
			lateral	53.9	8.0	1.9	39.3 - 68.2

Supplementary Table 12. Summary statistics corresponding to F-actin localization in Supplementary Fig. 14c. Information is presented as in Supplementary Table 1.

Location	Type of cell/Stage	# of cells	Membrane	Mean %	S.D.	S.E.M.	Range of %
proximal OC	*tbx5a* host 36 hpf	16	apical	16.0	5.8	1.5	6.6 - 24.7
			basal	38.7	12.2	3.1	20.0 - 61.0
			lateral	45.4	9.1	2.3	29.5 - 62.1
	tbx5a donor 36 hpf	3	apical	14.3	4.7	2.7	9.3 - 18.7
			basal	42.3	8.9	5.1	34.3 - 51.9
			lateral	43.4	4.2	2.4	38.8 - 47.0
distal OC	*tbx5a* host 36 hpf	21	apical	18.1	5.9	1.3	9.4 - 31.5
			basal	30.8	9.1	2.0	13.9 - 53.9
			lateral	51.2	8.9	1.9	36.1 - 67.8
	tbx5a donor 36 hpf	14	apical	20.8	9.0	2.4	6.2 - 30.9
			basal	30.5	14.2	3.8	7.4 - 60.1
			lateral	48.7	8.1	2.2	33.7 - 62.6
IC	*tbx5a* host 36 hpf	36	apical	20.0	9.5	1.6	5.6 - 50.6
			basal	34.5	14.1	2.3	13.7 - 72.0
			lateral	45.4	10.0	1.7	22.4 - 69.7
	tbx5a donor 36 hpf	14	apical	19.4	6.8	1.8	9.9 - 32.9
			basal	28.5	9.0	2.4	11.3 - 38.0
			lateral	52.2	5.6	1.5	42.7 - 64.0

Supplementary Table 13. Summary statistics corresponding to F-actin localization in Supplementary Fig. 14d. Information is presented as in Supplementary Table 1.
