# Supplementary material for: Regionalized regulation of actomyosin organization influences cardiomyocyte cell shape changes during chamber curvature formation
Source: Nat Commun. 2026 Mar 10;17:3768. doi: 10.1038/s41467-026-70384-5 (PMC13106787; doi:10.1038/s41467-026-70384-5)
Supplement: Supplementary file 2 — Reporting Summary [file 41467_2026_70384_MOESM2_ESM.pdf]

nature portfolio

Corresponding author(s): Deborah Yelon

Last updated by author(s): Dec 17, 2025

Reporting Summary

Nature Portfolio wishes to improve the reproducibility of the work that we publish. This form provides structure for consistency and transparency in reporting. For further information on Nature Portfolio policies, see our Editorial Policies and the Editorial Policy Checklist.

Statistics

For all statistical analyses, confirm that the following items are present in the figure legend, table legend, main text, or Methods section.

n/a	Confirmed	
☐	☒	The exact sample size (*n*) for each experimental group/condition, given as a discrete number and unit of measurement
☐	☒	A statement on whether measurements were taken from distinct samples or whether the same sample was measured repeatedly
☐	☒	The statistical test(s) used AND whether they are one- or two-sided<br>*Only common tests should be described solely by name; describe more complex techniques in the Methods section.*
☒	☐	A description of all covariates tested
☐	☒	A description of any assumptions or corrections, such as tests of normality and adjustment for multiple comparisons
☐	☒	A full description of the statistical parameters including central tendency (e.g. means) or other basic estimates (e.g. regression coefficient) AND variation (e.g. standard deviation) or associated estimates of uncertainty (e.g. confidence intervals)
☐	☒	For null hypothesis testing, the test statistic (e.g. *F*, *t*, *r*) with confidence intervals, effect sizes, degrees of freedom and *P* value noted<br>*Give P values as exact values whenever suitable.*
☒	☐	For Bayesian analysis, information on the choice of priors and Markov chain Monte Carlo settings
☒	☐	For hierarchical and complex designs, identification of the appropriate level for tests and full reporting of outcomes
☒	☐	Estimates of effect sizes (e.g. Cohen's *d*, Pearson's *r*), indicating how they were calculated

Our web collection on statistics for biologists contains articles on many of the points above.

Software and code

Policy information about availability of computer code

Data collection	Leica LAS X
Data analysis	Images were processed and analyzed in Imaris v11.0.0 and FIJI v2.16.0.<br><br>Statistics were performed in R (v4.4.1) and RStudio (v2025.09.1+401).<br>The following R packages were used to restructure, statistically analyze, and plot the quantitative data captured from micrographs: dplyr, ggbeeswarm, ggplot2, ggpmisc, ggpubr, ggtext, Lattice, plyr, Tidyverse, and ggnewscale.<br><br>Figures were produced in Adobe InDesign (v18.5.2.107) and Inkscape (v1.3.2).

For manuscripts utilizing custom algorithms or software that are central to the research but not yet described in published literature, software must be made available to editors and reviewers. We strongly encourage code deposition in a community repository (e.g. GitHub). See the Nature Portfolio guidelines for submitting code & software for further information.

Data

Policy information about availability of data

All manuscripts must include a data availability statement. This statement should provide the following information, where applicable:

- Accession codes, unique identifiers, or web links for publicly available datasets
- A description of any restrictions on data availability
- For clinical datasets or third party data, please ensure that the statement adheres to our policy

All source data is provided in the source data file.

Research involving human participants, their data, or biological material

Policy information about studies with human participants or human data. See also policy information about sex, gender (identity/presentation), and sexual orientation and race, ethnicity and racism.

Reporting on sex and gender	N/A
Reporting on race, ethnicity, or other socially relevant groupings	N/A
Population characteristics	N/A
Recruitment	N/A
Ethics oversight	N/A

Note that full information on the approval of the study protocol must also be provided in the manuscript.

Field-specific reporting

Please select the one below that is the best fit for your research. If you are not sure, read the appropriate sections before making your selection.

[x] Life sciences [ ] Behavioural & social sciences [ ] Ecological, evolutionary & environmental sciences

For a reference copy of the document with all sections, see nature.com/documents/nr-reporting-summary-flat.pdf

Life sciences study design

All studies must disclose on these points even when the disclosure is negative.

Sample size	Sample sizes were chosen based on recognized standards in the field of zebrafish development and previously published literature (e.g. PMID: 23533583, 31686119 and 35986301). We have reported the exact sample size for each experiment (number of replicates, embryos, and where appropriate, cells) in each corresponding figure legend. No statistical methods were used to predetermine the sample size.
Data exclusions	In figures where subcellular localization of F-actin or pMyosin is reported, statistically identified outliers with too high or too low overall signal intensity in the ventricular myocardium were not analyzed.
Replication	All experiments were verified with at least 4 biological replicates. In cases with multiple independent experiments, all replications were successful.
Randomization	For transplantation and transgenic plasmid injection experiments, all embryos were randomly assigned into experimental groups. For experiments using fluorescent transgenic lines, embryos were sorted for fluorescence before being randomly assigned into experimental groups. For experiments using mutants, embryos were sorted based on morphological phenotypes before being randomly assigned into experimental groups.
Blinding	Due to the inherent differences in morphology and/or fluorescent signal intensity between transgenics and wild-types and between mutants and wild-types, blinding was usually not possible. However, data analysis was performed using predefined protocols to reduce potential bias.

Reporting for specific materials, systems and methods

We require information from authors about some types of materials, experimental systems and methods used in many studies. Here, indicate whether each material, system or method listed is relevant to your study. If you are not sure if a list item applies to your research, read the appropriate section before selecting a response.

Materials & experimental systems

n/a	Involved in the study	
☐	☒	Antibodies
☒	☐	Eukaryotic cell lines
☒	☐	Palaeontology and archaeology
☐	☒	Animals and other organisms
☒	☐	Clinical data
☒	☐	Dual use research of concern
☒	☐	Plants

Methods

n/a	Involved in the study	
☒	☐	ChIP-seq
☒	☐	Flow cytometry
☒	☐	MRI-based neuroimaging

Antibodies

Antibodies used	The following primary antibodies were used at the specified dilutions: mouse anti-Alcama (Developmental Studies Hybridoma Bank, Zn-8 supernatant, 1:50); rabbit anti-Cdh2 (GeneTex, GTX125885, 1:200); rabbit anti-phospho-Myosin (Abcam, ab2480, 1:100); rabbit (Life Technologies, A11122, 1:500) or chicken (Life Technologies, A10262, 1:1000) anti-GFP; rabbit anti-dsRed (also detects mScarlet; Clontech, 632496, 1:1000); rabbit anti-TagRFP (also detects mKate; Evrogen, AB233, 1:500); mouse anti-myosin heavy chain (Developmental Studies Hybridoma Bank, MF20 supernatant, 1:50); and mouse anti-Myh6 (Developmental Studies Hybridoma Bank, S46 supernatant, 1:50).
Validation	- mouse anti-Alcama: PMID 32439760; https://dshb.biology.uiowa.edu/ZN-8<br>- rabbit anti-Cdh2: PMID 37083132; https://www.genetex.com/Product/Detail/Cdh2-antibody/GTX125885?srsltid=AfmBOor0-qB009XYKDDsmiOPgzCpBomG5aqHSvMMReTBiFnrGutZC8_g<br>- rabbit anti-phospho-Myosin: PMID 40053597; https://www.abcam.com/en-us/products/primary-antibodies/myl12a-phospho-s19-antibody-ab2480?sourceClicked=searchClickQuery-publications<br>- rabbit anti-gfp: PMID 32439760, 31444829; https://www.thermofisher.com/antibody/product/GFP-Antibody-Polyclonal/A-11122<br>- chicken anti-gfp: PMID 38293792, 28232600, 32439760; https://www.thermofisher.com/antibody/product/GFP-Antibody-Polyclonal/A10262<br>- rabbit anti-dsRed: PMID 28232600, 32439760; https://www.takarabio.com/products/antibodies-and-elisa/fluorescent-protein-antibodies/red-fluorescent-protein-antibodies?srsltid=AfmBOoqdqw0N6xXQ3EbGnQjJjDF3RxkUljAW0lAAMvGttgx4-btdPulG<br>- rabbit anti-TagRFP: PMID 23850773; https://evrogen.com/products/antibodies/AB-tRFP.shtml<br>- mouse anti-myosin heavy chain: PMID 17311471, 38293792, 28232600; https://dshb.biology.uiowa.edu/MF-20<br>- mouse anti-Myh6: PMID 37083132, 38293792, 28232600; https://dshb.biology.uiowa.edu/S46

Animals and other research organisms

Policy information about studies involving animals; ARRIVE guidelines recommended for reporting animal research, and Sex and Gender in Research

Laboratory animals	This study used Danio rerio embryos 50 hours post fertilization or younger.
Wild animals	N/A
Reporting on sex	Since sex-specific differentiation does not begin until late larval stages in zebrafish, we do not consider sex as a biological variable in the embryos used in this study.
Field-collected samples	N/A
Ethics oversight	All work presented here followed protocols (#S09125) approved by the Institutional Animal Care and Use Committee at the University of California, San Diego.

Note that full information on the approval of the study protocol must also be provided in the manuscript.

Plants

Seed stocks	N/A
Novel plant genotypes	N/A
Authentication	N/A
